# Overexpression of a Defensin Enhances Resistance to a Fruit-Specific Anthracnose Fungus in Pepper

**DOI:** 10.1371/journal.pone.0097936

**Published:** 2014-05-21

**Authors:** Hyo-Hyoun Seo, Sangkyu Park, Soomin Park, Byung-Jun Oh, Kyoungwhan Back, Oksoo Han, Jeong-Il Kim, Young Soon Kim

**Affiliations:** 1 Medicinal Nanomaterial Institute, BIO-FD&C Co. Ltd., Incheon, Korea; 2 Department of Biotechnology, Chonnam National University, Gwangju, Korea; 3 Experiment Research Institute, National Agricultural Products Quality Management Service, Seoul, Korea; 4 Biological Control Center, Jeonnam Bioindustry Foundation, JeollaNamdo, Korea; 5 Kumho Life Science Laboratory, Chonnam National University, Gwangju, Korea; Soonchunhyang University, Republic of Korea

## Abstract

Functional characterization of a defensin, J1-1, was conducted to evaluate its biotechnological potentiality in transgenic pepper plants against the causal agent of anthracnose disease, *Colletotrichum gloeosporioides*. To determine antifungal activity, J1-1 recombinant protein was generated and tested for the activity against *C. gloeosporioides*, resulting in 50% inhibition of fungal growth at a protein concentration of 0.1 mg·mL^−1^. To develop transgenic pepper plants resistant to anthracnose disease, *J1-1* cDNA under the control of 35S promoter was introduced into pepper via *Agrobacterium*-mediated genetic transformation method. Southern and Northern blot analyses confirmed that a single copy of the transgene in selected transgenic plants was normally expressed and also stably transmitted to subsequent generations. The insertion of T-DNA was further analyzed in three independent homozygous lines using inverse PCR, and confirmed the integration of transgene in non-coding region of genomic DNA. Immunoblot results showed that the level of J1-1 proteins, which was not normally accumulated in unripe fruits, accumulated high in transgenic plants but appeared to differ among transgenic lines. Moreover, the expression of jasmonic acid-biosynthetic genes and pathogenesis-related genes were up-regulated in the transgenic lines, which is co-related with the resistance of J1-1 transgenic plants to anthracnose disease. Consequently, the constitutive expression of J1-1 in transgenic pepper plants provided strong resistance to the anthracnose fungus that was associated with highly reduced lesion formation and fungal colonization. These results implied the significance of the antifungal protein, J1-1, as a useful agronomic trait to control fungal disease.

## Introduction

Higher plants have innate defense systems to protect themselves against biotic stresses [1]–[3]. A range of protective molecules, including antimicrobial proteins, are synthesized in the tissues invaded by pathogens or accumulated during normal growth [4]–[6]. Defensins that belong to antimicrobial peptide superfamily are a large class of small peptides occurring in various living organisms, ranging from microorganisms to plants and mammals [7], [8]. On the basis of structural and functional similarity with insect defensin, plant antimicrobial peptide called γ–thionin in wheat and barley grains was renamed as defensin [9]. Plant defensins are composed of three anti-parallel β-strands and one α-helix with a characteristic three-dimensional folding stabilized by four disulfide bonds [10]. The cysteine-stabilized α-helix/β-sheet (CSαβ) motif confers great stability on the peptide to maintain the functional activity [11].

The main biological function of plant defensins was found to inhibit the growth of a broad range of phytopathogenic fungi at micromolar concentrations [12]. Other biological activities of defensins have also been proposed as protein synthesis inhibitors, α-amylase inhibitors, zinc tolerance mediators, and ion channel blockers [13]–[16]. Although the action mode of plant defensin in fungal growth inhibition has not been clearly understood, the inhibition of fungal growth is followed by initial binding of the defensin on fungal membrane due to electrostatic and/or hydrophobic interactions. Indeed, a higher concentration of defensins causes severe membrane permeabilization, which leads to fungal death [17]–[20]. However, this arouses a controversy that the peptides could disrupt the integrity of membranes not only in the fungal cells, but also in plant cells. Regarding the localization, plant defensins were generally predicted to be secreted to extracellular space due to the occurrence of signal peptide at their N-terminal. Previously, subcellular localization analysis showed that several plant defensins were deposited to cell wall [9]. Otherwise, a flower defensin, NaD1, has been immunolocalized in the vacuole in *Nicotiana alata* [21], and AhPDF1.1 from *Arabidopsis halleri* was retained in internal compartments while moving to the lytic vacuole [22]. Therefore, further studies are necessary to clarify the action mode in association with the localization of defensins.

Cumulative studies demonstrate that defensins are expressed in various vegetative tissues of plant [23], [24]. In addition, reproductive organs, such as floral organs or fruits, express defensins as part of a predetermined developmental program or induce defensins under stressed conditions associated with the invasion of a pathogen [25], [26]. A defensin, designated as J1-1, has been previously described in the fruit of bell pepper [27]. Expression of the *J1-1* gene was found to occur during the ripening of the fruit and was also inducible by wound or pathogen. Thus, J1-1 was suggested to play a role in protecting the reproductive organs against biotic and abiotic stresses. *In vitro* antifungal assay has shown that J1-1 protein isolated from ripe pepper fruits effectively suppressed the mycelial growth of *Fusarium oxysporum* and *Botrytis cinerea*. However, further questions about the functional activity of the protein in the infected pepper cells need to be addressed. For example, although the expression of the *J1-1* gene in ripe fruit during an incompatible interaction with *Colletotrichum gloeosporioides* provided indirect evidence for the role of J1-1 in protecting the ripe fruits against pathogen attack [28], genetic and biological studies of J1-1 in relation to disease resistance have been lacking.

Chilli peppers have been cultivated worldwide and are one of the most important vegetable fruits in some areas. Major constraints to the pepper fruit production include pests and diseases, and anthacnose disease is the most notifiable infectious disease caused by *Colletotrichum* species [29], [30]. Immature pepper fruits in green color are vulnerable to the pathogen, causing widespread outbreaks of the disease. Despite large-scale breeding efforts to control the anthacnose disease, it remains as an endemic disease, resulting in large reductions of annual yields worldwide [31]. Thus, it is necessary to build up novel genetic resources for the development of anthracnose disease-resistant peppers, and genetic engineering is a feasible approach to generate anthracnose-resistant pepper plants [32]. Although constitutive expression of plant defensins has shown enhanced resistance in tomato, potato, and canola against various pathogens [33]–[37], anthracnose disease-resistant plants have not yet been developed.

In this study, we investigated the antifungal activity of J1-1 protein against the anthracnose fungus and the localization of the peptide in infected fruits. The characteristics of the pepper defensin prompted us to develop transgenic pepper plants and to assess the contribution of the defensin to pepper resistance against the anthracnose fungal infection using the transgenic pepper plants overexpressing J1-1. The results suggest that J1-1 is an attractive candidate for biotechnological application to provide enhanced resistances in pepper, especially to the anthracnose fungus.

## Materials and Methods

### Plant Materials and Growth Conditions

*Capsicum annuum* cv. Nokkwang was used in plant transformation experiments, as previously described [38]. Wild-type and transgenic seedlings were grown in a growth chamber at 25°C and 50% humidity in a light/dark cycle of 16/8 h. Pepper plants were transferred to soil and grown in a greenhouse for further experiments. Samples were collected from 2-month-old plants, except for ripening fruits. Mature fruits were harvested at the following ripening stages: stage I, green fruit; stage II, early breaker fruit; stage III, turning fruit; stage IV, purple fruit; stage V, red fruit.

### Fungal Pathogens and Inoculation

The monoconidial isolate KG13 of *C. gloeosporioides*, which is only compatible with unripe pepper fruits, was used to elucidate the functional role of J1-1 protein in infected unripe fruits [39]. Growth and spore harvests of the fungus were performed as described previously [40]. Fungal inoculation was conducted by applying a drop of spore suspension (density of 5×10^5^ spores per 1 mL in distilled water) onto mature unripe green fruits. The inoculated fruits were placed in high humidity and dark conditions for 1 day to stimulate infection. Thereafter, the fruits were incubated at 26°C in a growth chamber until harvesting. For analysis of the J1-1 protein, a piece (5×5 mm) of pericarp was taken from the inoculated sites of the fruits at 0, 3, 24, 48, and 72 hours.

The growth of the fungus on infected fruits was analyzed using the non-transformed or transgenic unripe fruits after fungal infection. For microscopic observation, 0.1% toluidine blue was topically applied on the infection area of the fruits at one day after infection and then peeled the skin off to observe the fungus under microscope. At five days after infection, transversal sections of the fruits were stained with lactophenol trypan blue to visualize the fungal hyphae. In addition, the development of anthracnose symptom was monitored until 9 days after infection. Then, disease rate was expressed as percentage of the number of lesions from infected spots. The sporulation was determined by counting the number of spores from a lesion.

### *In vitro* Antifungal Activity Assay with Recombinant J1-1 Protein

The cDNA encoding *J1-1* was cloned into pGEX-6P-1 (Amersham Biosciences, Freiburg, Germany) between *Eco*RI and *Xho*I, creating an in-frame fusion with the sequence encoding glutathione-S-transferase (GST). The primers used were a forward primer (5′-GGAATTCCTTATGGCTGGCTTTTCCAAAG-3′) and a reverse primer (5′-CCCTCGAGGGATTAAGCACAGGGCTTCGT-3′). The GST fusion protein was then expressed in *E. coli* strain BL21 and purified according to the manufacturer's instructions. The protein concentration was determined using the Bradford method. Following purification, the antifungal activities of the GST/J1-1 fusion protein were examined against *C. gloeosporioides.* The fungal growth was monitored by microscopic examination on cover glass with 5×10^2^ spores in sterile water containing various concentrations of GST/J1-1 recombinant protein or heated protein obtained by incubating at 90°C for 10 min. The spores were treated with the proteins for 24 hours at 26°C, and then counted for germination and appressorium formation in at least five microscopic fields. The experiment was conducted in triplicate.

### Plasmid Construction and Pepper Transformation

A full-length cDNA of *J1-1* was amplified by PCR using the primers, 5′-GCTCTAGAGCATGGCTGGCTTTTCCAAAG-3′ (forward) and 5′-CGGATCCGTTAAGCACAGGGCTTCGT-3′ (reverse). The resulting fragment was cloned between *Xba*I and *Bam*HI in pBI121. Then, the expression cassette spanning the CaMV 35S promoter to the Nos terminator was transferred into pCAMBIA1300 between *Hin*dIII and *Eco*RI sites. The pCAMBIA1300/*J1-1* was mobilized into *Agrobacterium tumefaciens* GV3101 and used to generate transgenic pepper plants.

The cotyledon and hypocotyl explants were inoculated with *Agrobacterium* suspensions as described previously [38]. Following infection, the regeneration of the primary transformants was accomplished on selection medium containing 20 mg·L^−1^ hygromycin and 300 mg·L^−1^ cefotaxime. Plantlets resistant to hygromycin were then transferred onto rooting medium containing MS basal salts supplemented with 300 mg·L^−1^ cefotaxime. All cultures were incubated at 26°C under a 16/8 hr (light/dark) photoperiod. Plants having well-developed roots were transplanted to pots and grown in a greenhouse until they flowered. Primary transgenic plants (T_0_) were self-pollinated and their seeds (T_1_) were germinated in MS medium containing 20 mg·L^−1^ hygromycin. The number of green seedlings resistant to hygromycin was counted. The data were then analyzed using the χ^2^ test to determine the number of functional *HPT* gene loci on the pepper genome. Self-pollinated T_2_ progenies were also tested for hygromycin resistance to identify homozygosity. From these procedures, four transgenic pepper lines were generated and used for further analysis.

### Southern and Northern Blot Analyses

To analyze the genomic DNA for integration of the *J1-1* gene, pepper genomic DNA was isolated using a DNeasy Plant Maxi Kit (Qiagen, Hilden, Germany) as described by the manufacturer. For Southern blot analysis, 15 µg of each DNA sample was digested with *Eco*RI and separated on a 1.0% (w/v) agarose gel. The digested DNA was then transferred to a nylon membrane and hybridized with *HPT* or *J1-1* gene probes that was labeled with [α^32^P] dCTP using the Rediprime II Random Prime Labeling System (Amersham Biosciences, UK). After hybridization, the membranes were exposed at −80°C on Kodak XAR-5 film (Kodak, Rochester, NY) using an intensifying screen.

For Northern blot analysis, total RNA was extracted from the pepper fruits using a RNeasy Plant Kit (Qiagen, Hilden, Germany). 10 µg of the total RNA was separated on 1.2% denaturing agarose gels and blotted onto a Hybond N^+^ membrane (GE Healthcare, Buchinghamshire, UK). The blots were then hybridized with [α^32^P] dCTP-labeled respective probes that were amplified by PCR. The primers used for probes were shown in Table S1.

### Immunoblot Analysis and J1-1 Antibody Production

The samples were homogenized in an extraction buffer (50 mM Tris, pH 8.0, 2 mM EDTA, 2 mM DTT, 0.25 M sucrose and protease inhibitor cocktail (Roche, Mannheim, Germany)) at cold conditions and subjected to centrifugation at 3000 g for 15 min. The supernatant was used as a total protein. For immunoblot analysis, total proteins were separated by 12% SDS-PAGE and transferred onto polyvinylidene fluoride (PVDF) membranes. A dilution of polyclonal anti-J1-1 rabbit antibody was used for immunoblot analysis, which was followed by peroxidase-conjugated anti-rabbit antibody. The anti-J1-1 serum was raised against a KLH-conjugated peptide corresponding to amino acid sequences (26-39 AA; KICEALSGNFKGLCL) of J1-1 as described previously [27].

### Immunohistochemical Localization of J1-1 Protein

For immunolocalization, pepper fruits were fixed in 0.1% glutaraldehyde and 4% paraformaldehyde in a 50 mM sodium phosphate buffer (pH 7.0), dehydrated in ethanol, and embedded in paraffin. Tissues were sliced into 5-µm thick transverse sections. The deparaffinized sections were incubated with anti-J1-1 antibody (1:2000) for 4 h at 12°C, followed by incubation with peroxidase-conjugated anti-rabbit antibody according to the manufacturer's instruction (DAKO, Carpinteria, CA). Control experiments using pre-immune serum were not reactive (data not shown).

### Inverse PCR Analysis

The genomic DNA (gDNA) sequences flanking a T-DNA insertion were cloned from the transgenic lines using inverse PCR (i-PCR). gDNA was isolated from the leaves of independent T_2_ transgenic pepper plants and digested with a restriction enzyme that was chosen in the T-DNA region of the pCAMBIA1300/*J1-1* binary vector with unique restriction sites, such as *Bgl*II, *Eco*RI, or *Hin*dIII. After purification, 1 µg of gDNA was self-ligated in a 250-µL reaction volume using 40 units of T_4_ DNA ligase (Promega, Madison, WI). The circularized DNA was purified, and 100 ng of DNA was used as a template for i-PCR reactions. Two sets of primers, which were specifically designed for the sequences of T-DNA and the *J1-1* gene, were sequentially used: IP-F1/IP-R1 and IP-F2/IP-R2 for right border (RB), and IP-F3/IP-R3 and IP-F4/IP-R4 for left border (LB) (Table S1). The PCR condition was 5 min at 94°C, 35 cycles of 94°C for 30 sec, 58°C for 30 sec, and 72°C for 2 min with a 10-min extension period at 72°C. The resulting PCR products were cloned into a TOPO vector (Invitrogen, Carlsbad, CA) and subjected to sequencing. The gDNA sequences were compared using the basic local alignment search tool (BLAST).

### Statistical analysis

Experimental data were subjected to analysis of variance (ANOVA) using IBM SPSS statistics 20 software. Significant difference of mean values was compared by the LSD and DMRT at *P<0.05*. All of the data were represented as the mean ± SD of at least three independent experiments.

## Results

### Fruit-specific Accumulation of J1-1 Protein upon Ripening and Its Inducibility by Fungus

**Figure 1:**
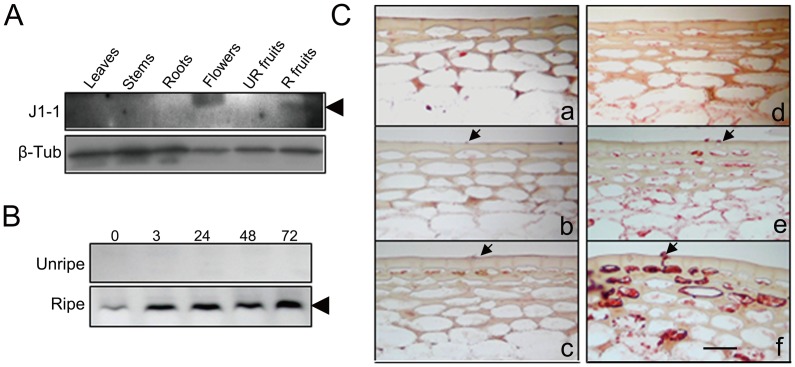
Expression of J1-1 is related to fruit ripening and induced by fungal infection in pepper fruits. **A,** Organ-specific expression of J1-1 protein in leaves, stems, roots, flowers, unripe (UR) and ripe (R) fruits of pepper. β-tubulin was shown as a loading control. An arrowhead indicates the protein band of J1-1. **B,** Fungal-induced J1-1 accumulation in unripe and ripe pepper fruits infected with *C. gloeosporioides*. Numbers on the top represent hours after infection (HAI). Immunoblot analysis was performed with total soluble proteins from pepper tissues using polyclonal J1-1 antibody. **C,** Immunolocalization of J1-1 in unripe (**a**–**c**) and ripe (**d**–**f**) fruits at 0, 24, and 48 h after inoculation. To localize the protein, transverse sections of pepper fruits were incubated with polyclonal J1-1 antibody that was detected with AEC (3-amino-9-ethylcarbazole) chromogen, shown as red. The arrows indicate fungal spores on the surface of the pepper fruits. Bar  = 50 µm.

To understand how the expression of J1-1 is associated with fruit development, immunoblot analysis was used to compare the expression levels in various pepper tissues. J1-1 was not detected in non-fruit tissues such as leaf, stem, root, flower and unripe green fruit, but was detected in the ripe red fruit (Figure 1A and S1). J1-1 protein was gradually increased in the fruits from the early stage of the ripening, indicating the developmental regulation of the fruit-specific expression of J1-1 (Figure S2). Additionally, the presence of a higher band which is detected in the flower suggests the occurrence of another defensin member. Then, the induction of the J1-1 was monitored in the pepper fruits infected with the fruit-specific fungal pathogen, *C. gloeosporioides*. As shown in Figure 1B, J1-1 was not detected in infected unripe fruits, even though expression of the gene at the transcriptional level was previously reported in fruits in response to a pathogen [28]. On the contrary, the level of the J1-1 protein was increased at 3 hours after infection (HAI) in the ripe fruit and was maintained during the period of observation. These results suggest that differential regulation of the J1-1 expression likely occurred at both transcriptional and translational levels in infected fruits during ripening.

Moreover, immunolocalization analysis using J1-1 antibody demonstrated that the consistent expression of the J1-1 protein occurred in healthy ripe fruit (Figure 1C). J1-1 protein was not detected in unripe fruit, even around the infection site undergoing necrotized cell death in the epidermis (Figure 1C-c). In contrast, the protein was evenly expressed in the pericarp of the ripe fruit (Figure 1C-d). In response to fungal attack, a strong positive signal was noted near the position where the fungus had broken into the cell wall of the epidermis of ripe fruit. Figure 1C-f shows that the protein was secreted to the outside of epidermal cells. The protein was also highly detected in the cytoplasm of some cells around the infected area. In the negative control with preimmune serum, no positive signal was detected in the tissues (data not shown). The results suggest that the J1-1 protein is preferentially accumulated in the peripheral cell layers of the ripe fruits and secreted to the invading pathogen, indicating the role in the first line of plant defense against pathogen attack.

### Antifungal Activity of J1-1 Recombinant Protein

**Figure 2:**
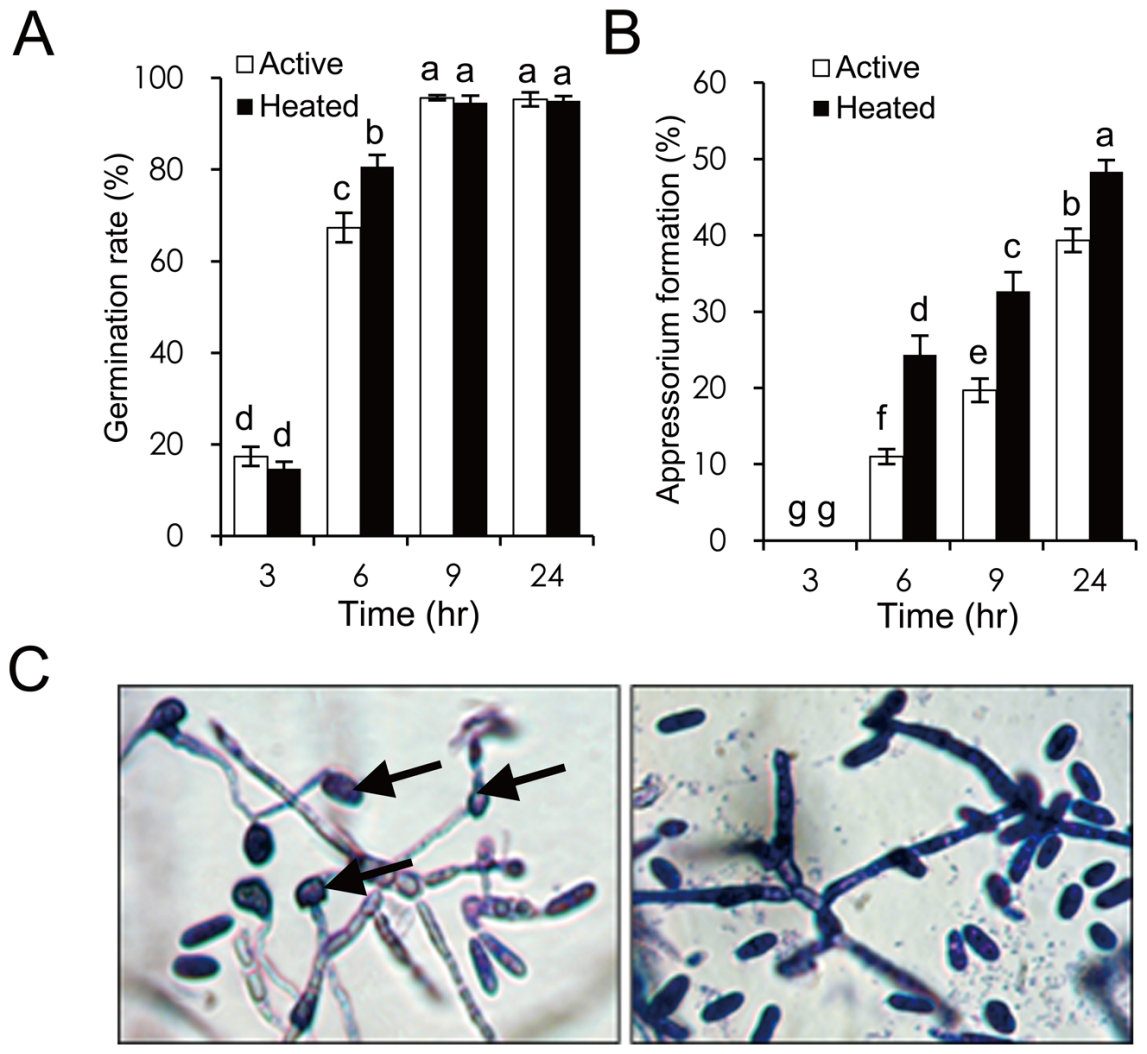
J1-1 recombinant protein shows antifungal activity against *C. gloeosporioides*. **A,** Spore germination. **B,** Appressorium formation. Spore suspensions were amended with 10 µL of the GST/J1-1 recombinant protein or its heated protein to final concentrations of 0.1 mg·mL^−1^. The protein was heated by incubating at 90°C for 10 min. A minimum of 100 spores were counted per replicate. Each value represents the mean ± SD of three replicates. Means with different letters in each column are significantly different at P<0.05. **C,** Representative photos of fungi that were treated with 0.1 mg·mL^−1^ of GST/J1-1 recombinant protein for 48 hours (right). Control was treated with distilled water (left). Arrows indicate appressorium.

To assess the possible function of J1-1, the antifungal activity of J1-1 protein was examined against a major fruit pathogen that causes sunken disease in unripe fruits. Its effect was evaluated according to the spore germination and appressorium development of *C. gloeosporioides*. For microscopic observation, 10 µL of spores diluted in sterile water to a density of 5×10^5^ mL^−1^ was mixed with GST/J1-1 protein to yield mixtures of 0.001, 0.01, 0.1, and 1 mg·mL^−1^ on cover glass and kept in a humidified chamber at 26°C for 24 hours. The results showed that the 0.1 mg·mL^−1^ mixture of GST/J1-1 protein had a 50% inhibitory effect on appressorium formation from the germination tube of the fungus (Figure 2). However, spore germination was barely affected regardless of the protein concentration (Figure S3). When the protein was heated by incubating at 90°C for 10 min, the growth inhibition was reduced by approximately 25% compared with active J1-1. These results indicate the activity of J1-1 against the pathogenic fungus, *C. gloeosporioides*, and also suggest the potential of J1-1 for plant disease control in economically important pepper cultivation.

### Development of Stable Transgenic Pepper Plants Carrying *J1-1*

**Figure 3:**
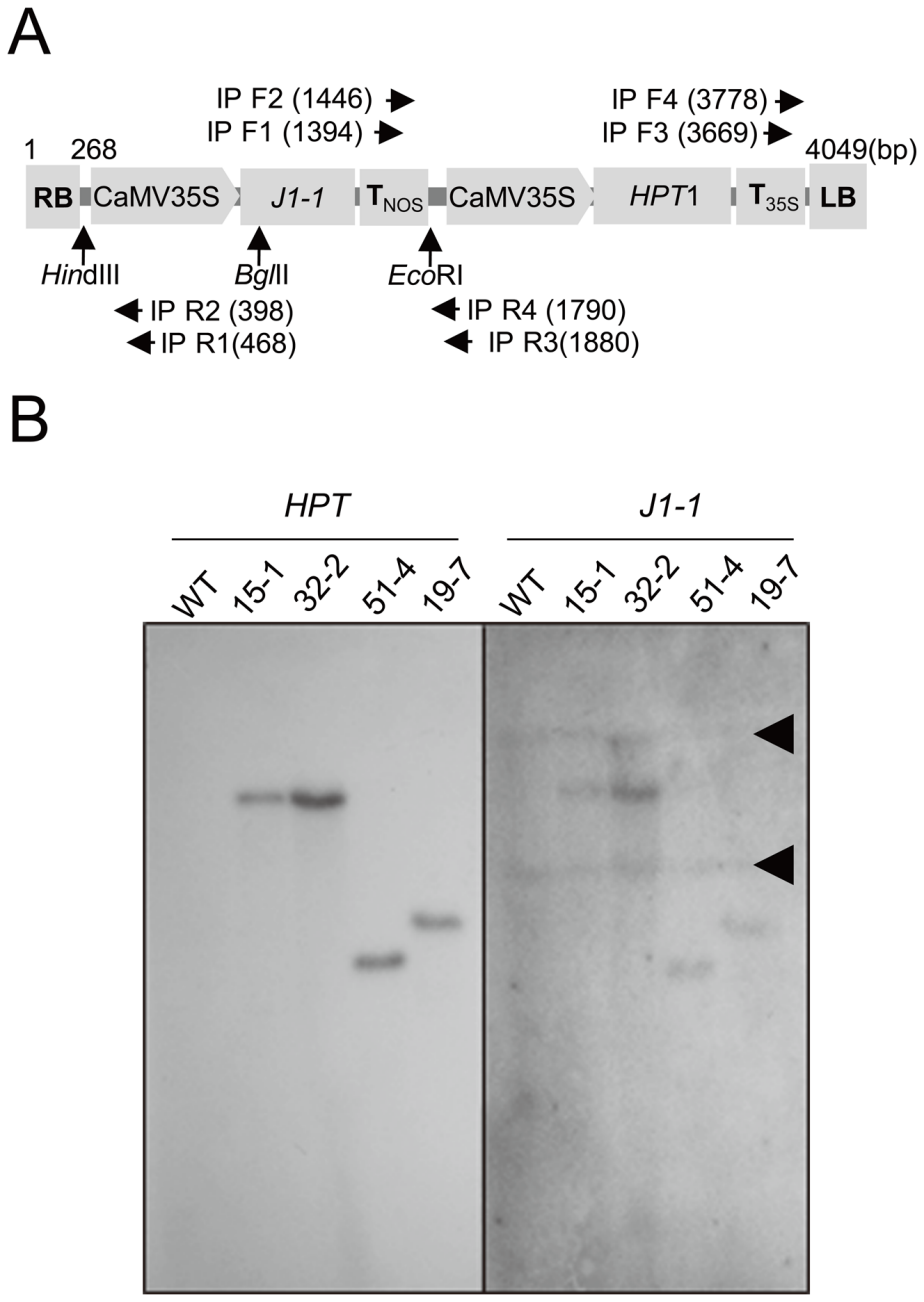
Southern blot analysis of transgenic pepper plants carrying *J1-1* gene. **A,** Schematic diagram of the T-DNA representing restriction enzyme sites and primer sites for i-PCR. LB, T-DNA left border repeat; RB, T-DNA right border repeat; *HPT1*, hygromycin phosphotransferase I; CaMV35S, CaMV 35S promoter; T_NOS_, transcriptional terminator of nopaline synthase (*NOS*); T_35S_, CaMV 35S transcriptional terminator. **B,** Southern blot analysis. gDNA was digested with *Hin*dIII, and hybridized with ^32^P-labeled *HPT1* probe (left) or rehybridized with the *J1-1*gene (right). WT, non-transformed wild-type pepper plant. Arrowheads indicate endogenous J1-1 bands.

Based on the proposed function of J1-1, transgenic pepper plants were generated to express the *J1-1* gene under the control of the CaMV 35S promoter and nopaline synthase transcriptional terminator (Figure 3A). Cotyledonary explants were infected with *Agrobacterium* cells harboring pCAMBIA1300/*J1-1* as described previously [38]. During subcultures of explants, 20 mg·L^−1^ hygromycin was used for the callus induction and 10 mg·L^−1^ was used at the regeneration stage to select transgenic shoots. Adventitious buds were transferred to rooting media, in which putative transgenic plants were produced (Figure S4). Since these plants displayed normal phenotypes in the pots, their seeds were obtained from self-pollination. Of the nine primary transformants, eight were identified as containing T-DNA using preliminary PCR screening with the combination of a sequence from the 35S CaMV promoter as a forward primer and a sequence from the *J1-1* cDNA as a reverse primer (data not shown).

To determine whether the transgenes were stably inherited to the next generation, seeds harvested from eight primary transgenic pepper lines were evaluated for resistance to 20 mg·L^−1^ hygromycin. Segregation ratios of 2.4∼3.8:1 were observed in four lines and χ^2^ analysis verified a 3:1 segregation for the *HPT* gene, indicating Mendelian segregation of a single dominant gene (Table S2). The results also suggest that transgenic pepper plants carrying the *J1-1* gene were genetically stable in advanced generations. Thus, after analyzing the antibiotic resistance of T_2_ seeds, four homozygous lines carrying a single copy of T-DNA were selected and used in the subsequent experiments.

### Molecular Characterization of Transgenic Pepper Plants

Stable integration and expression of the transgenes was further investigated in four selected T_1_ progenies. Southern blot analysis was conducted with genomic DNA isolated from J15-1, J32-2, J51-4, and J19-7 plants, as well as non-transformed wild-type (WT) plant as a negative control (Figure 3B). The genomic DNAs were digested with *Hin*dIII and hybridized with a probe composed of the *HPT* gene. While genomic DNA from control plant (WT) showed no hybridization signal to the probe DNA, each primary transgenic line exhibited a single band with a different band pattern, except for J32-2 which exhibited the same band mobility as J15-1. The result indicates that the J15-1/J32-2, J19-7, and J51-4 plants were independent events of transformation. To confirm the integration of *J1-1* in the transgenic plants, the membrane was deprobed and rehybridized with the *J1-1* gene as the probe. The result demonstrated that each transgenic plant showed the same band patterns corresponding to the *HPT* band (Figure 3B). In addition, two endogenous *J1-1* bands were detected in all lanes including the control plant. Since the T-DNA of pCAMBIA1300/*J1-1* has a unique *Hin*dIII site, the result indicates that a single copy of the *J1-1* gene along with *HPT* gene was integrated into the pepper genome.

**Figure 4:**
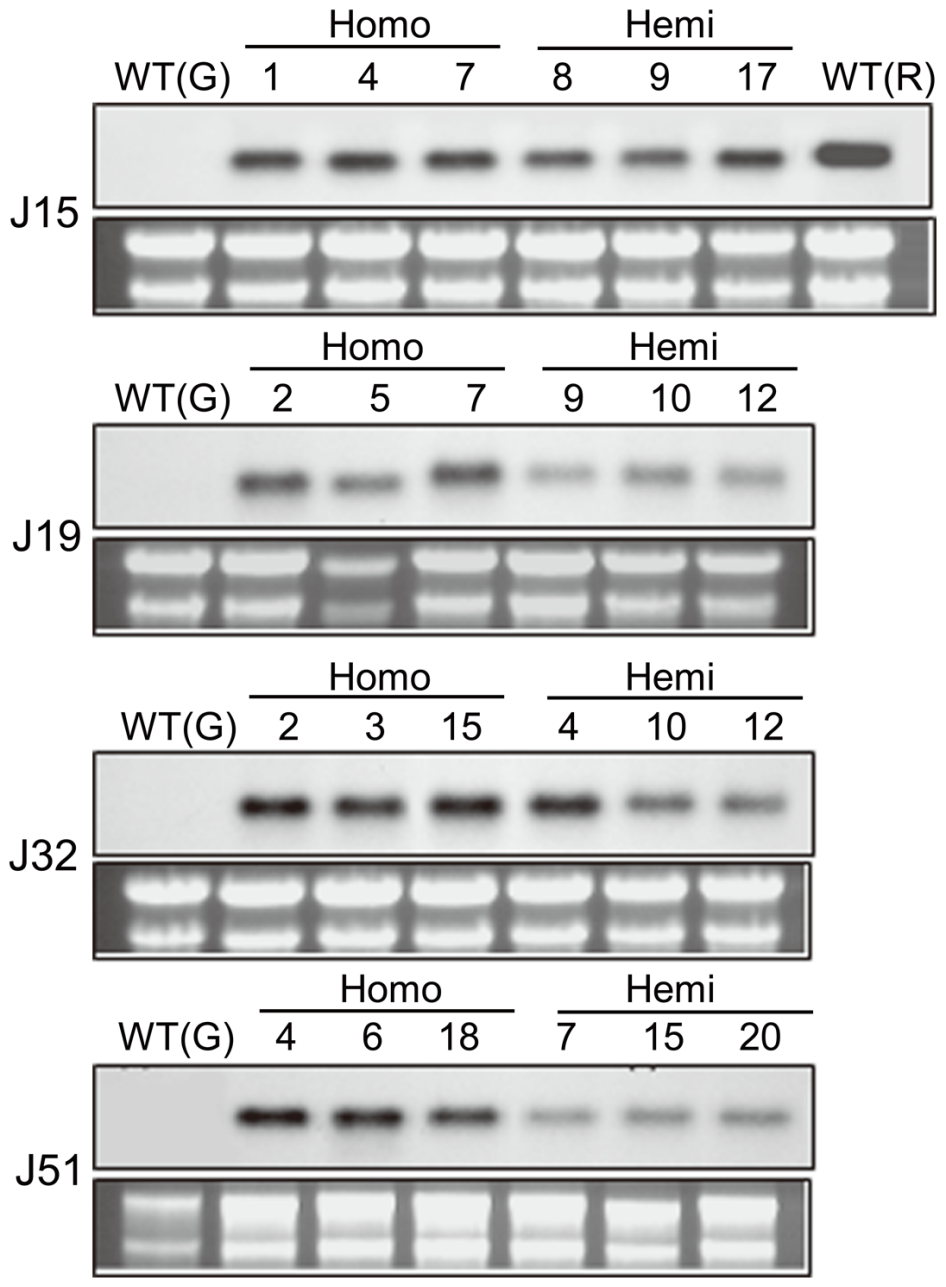
Northern blot analysis of unripe fruits from transgenic pepper lines. Lane 1, green fruit (G) from wild-type (WT) plant as a negative control; lanes 2–4, three T_1_ transgenic plants representing homozygous progenies; lanes 5–7, three T_1_ transgenic plants representing hemizygous progenies; lane 8, ripe fruit (R) from WT plant as a positive control. ^32^P-labeled *J1-1* was used as a probe, and total RNAs were shown as loading controls in lower panels.

Northern blot analysis was carried out using four transgenic T_1_ plants to confirm stable expression of the introduced transgene in the transgenic pepper plants. Total RNA was extracted from three homozygous and hemizygous progeny plants from genetically independent T_1_ plants and hybridized with a *J1-1* cDNA probe. At the mRNA level, the introduced *J1-1* gene was transcriptionally active in the unripe fruits of the transgenic lines, as well as in the wild-type ripe pepper fruit as a positive control, while no signal was detected in the unripe fruits of the non-transformed control plant (Figure 4). In general, homozygous plants showed higher levels of transgene expression, while a hemizygous state led to weak expression in the transgenic plants carrying a single copy of T-DNA. These results indicate that the introduced gene was stably expressed, but the expression level was dependent on the hemizygosity of the transgene in the transgenic progenies.

To understand the correlation between T-DNA integration and its genetic stability, we cloned and sequenced the genomic DNA flanking both the RB and LB of T-DNA in each transgenic line. Two consecutive primers were designed in the vicinity of a unique restriction enzyme, such as *Eco*RI, *Hin*dIII or *Bgl*II, in the T-DNA region (Figure 3A). Genomic DNA was extracted from three independent transgenic pepper lines and digested with an appropriate enzyme to clone right and left T-DNA/gDNA junctions. After self-ligation of the gDNA, the ligated DNA was used as a template for i-PCR with a pair of primers; IP F1/IP R1 and IP F3/IP R3 were typically used for the RB and LB, respectively. As summarized in Table 1, five events from either side of the T-DNA revealed that deletion of a short DNA fragment ranging from 52 bp occurred in the border sequence of the T-DNA integrated in the pepper genome. There were two instances of more extensive deletion on the left border of T-DNA: 344 bp in J15 and 289 bp in J19 transgenic plants (Table S3). In transgenic line J15, the deleted fragment at the LB included 28 bp in the C-terminal of the *hygromycin phosphotransferase* (*HPT1*) gene, which resulted in an impairment of the original stop codon that caused an additional tail with 20 amino acids. However, the J15 transgenic progenies were capable of retaining antibiotic resistance against hygromycin. The results indicate that the genetic stability of the *HPT* gene was maintained in all transgenic pepper lines in spite of the exclusion of the T-DNA junction fragments. In addition, gDNA sequence analysis of the T-DNA insertion sites of three transgenic lines did not reveal any similarity compared with known EST or gDNA sequences in the public database.

**Table 1:** Sequence analysis of T-DNA/gDNA junctions in transgenic pepper lines by i-PCR.

Line	Border	Enzyme^a^	gDNA (bp)^b^	Deletion (bp)^c^	Primers
J15	RB	*Eco*RI	1961	52	IP F1/IP R1
	LB	*Bgl*II	951	344	IP F4/IP R3
J19	RB	*Eco*RI	98	63	IP F1/IP R1
	LB	*Hin*dIII	1581	289	IP F3/IP R2
J51	RB	*Eco*RI	ND	ND	IP F1/IP R1
	LB	*Eco*RI	1776	55	IP F3/IP R3

ND, not determined.

a Restriction enzyme used for gDNA rescue.

b Length of rescued gDNA flanking the T-DNA border.

c Deleted length at endpoint of the T-DNA.

### *J1-1* Transcript and Protein Accumulation in Transgenic Pepper Plants

**Figure 5:**
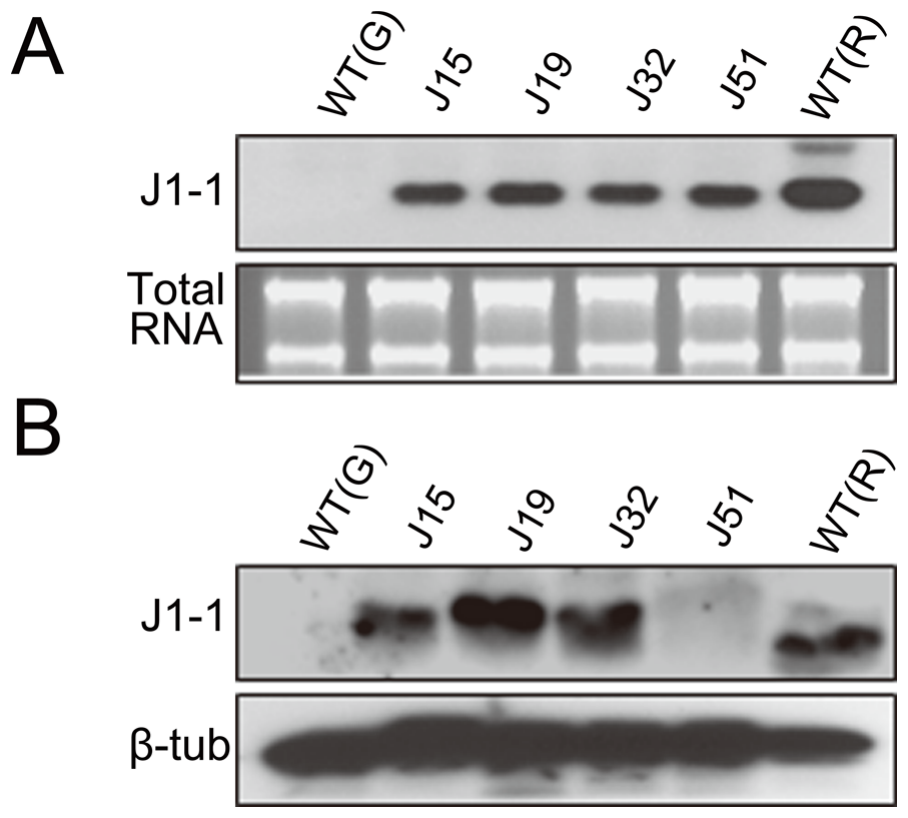
Expression of the J1-1 in the unripe pepper fruits of transgenic plants. **A,** Northern blot analysis of the *J1-1* transcript. Total RNA from each T_2_ progeny was hybridized to a radiolabeled *J1-1* probe. Lane 1, unripe green fruits (G) of non-transformed wild-type (WT) pepper plant as a negative control; lanes 2–5, four T_2_ transgenic lines representing homozygous progenies; lane 6, ripe fruits (R) of non-transformed pepper as a positive control. **B,** Immunoblot analysis of the J1-1 protein. Total soluble proteins from T_2_ progenies were subjected to immunoblot analysis with polyclonal anti-J1-1 antibody. Total RNA and β-tubulin were shown as loading controls.

Since unripe green fruits are extremely vulnerable to infection by the hemibiotrophic anthracnose fungus, we generated transgenic plants overexpressing J1-1 constitutively to control the disease. Before investigating fungal resistance of the transgenic pepper plants, the expression of J1-1 was initially examined in the unripe fruits of transgenic plants (T_2_) at both mRNA and protein levels, as compared with that in wild-type ripe fruit as a positive control. The results showed that a considerable amount of the *J1-1* transcript was detected in the unripe green fruits of the transgenic lines, but not detected in those of the non-transformed control plant (Figure 5A). Marked accumulations of the J1-1 proteins were also observed in the green fruits of the transgenic lines except line J51, which revealed a slightly detectable amount of J1-1 protein (Figure 5B). From detailed observation with the J32 and J51 transgenic lines, a strong band of the *J1-1* transcript was detected in the unripe fruits of the both lines, indicating that the mRNA expression is normal in the J51 line (Figure S5A). Moreover, the expression of the *J1-1* transcript was increased in both lines at 24 hours after inoculation (HAI) with *C. gloeosporioides*. Unlike mRNA expression, J1-1 protein was detectable in J51 line only after fungal infection at 24 HAI, whereas J32 showed highly accumulated J1-1 protein in both healthy and infected fruits (Figure S5B). In the non-transformed control plants, a slight increase in *J1-1* mRNA was shown in the unripe fruit at 24 HAI, but the fruit showed no induction of its protein. This result suggests that J1-1 protein might be unstable in healthy unripe fruits while the transcript was durable.

### Expression of Jasmonic Acid (JA) Biosynthetic and Pathogenesis–Related (PR) Genes in J1-1 Transgenic Pepper Fruits

**Figure 6:**
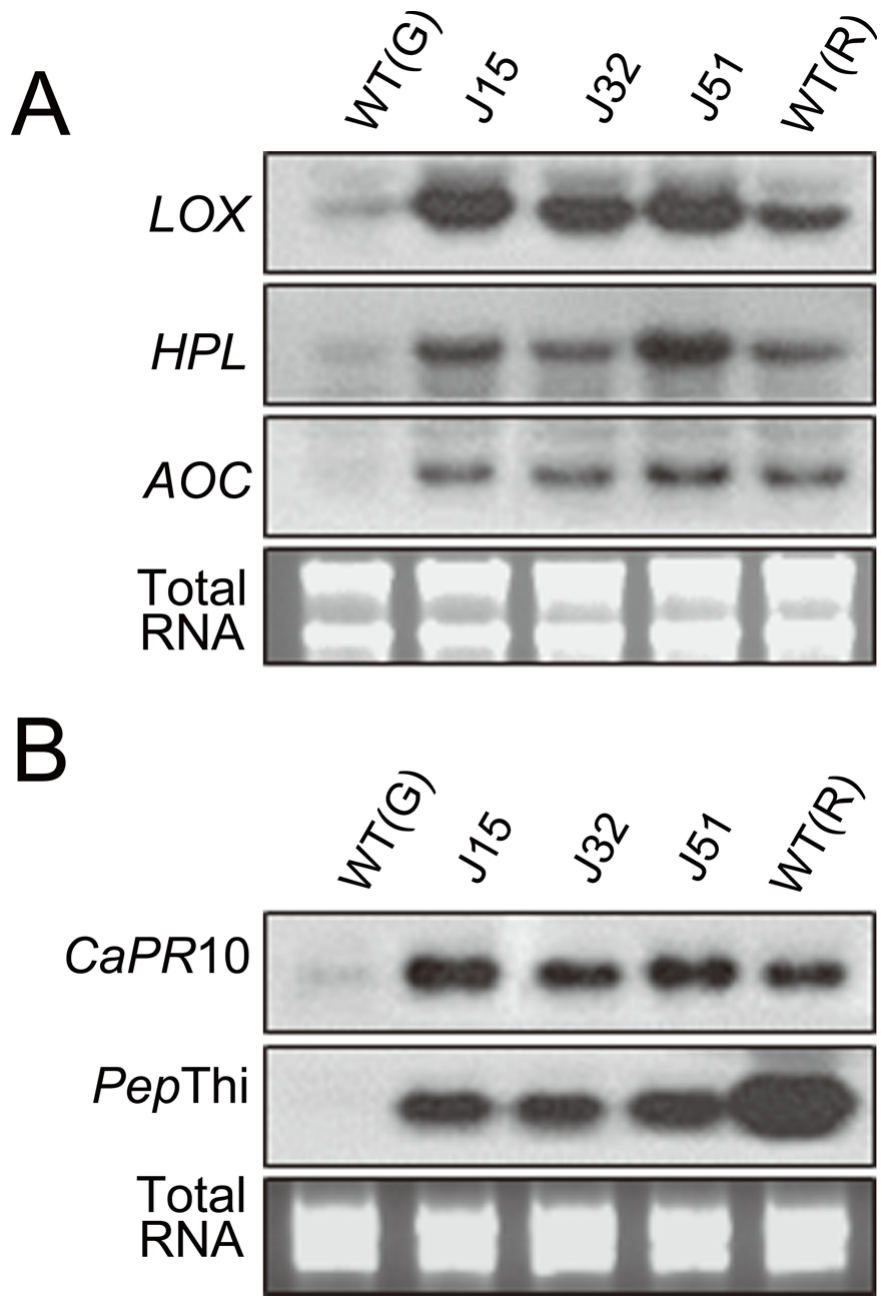
Expression of JA-biosynthesis related genes (A) and pathogenesis-related genes (B) in transgenic pepper fruits. Total RNAs were extracted from the unripe fruits of T_2_ transgenic pepper lines (J15, J32, and J51). 10 µg of total RNA was separated in a formaldehyde/agarose gel, transferred onto nylon membrane, and hybridized to radiolabeled respective probes. WT (G), non-transgenic unripe fruits as a negative control; WT (R) non-transgenic ripe fruits as a positive control.

Previously, the expression of *J1-1* gene was shown to be up-regulated in the unripe and ripe pepper fruits by exogenous treatment of methyl jasmonate [28]. In addition, it has been reported that endogenous level of JA rises with the onset of ripening in several fruits [41]. Thus, it could be expected that JA signaling involves in J1-1 induced disease resistance in pepper fruits. In the present study, the expression of JA biosynthetic genes, such as lipoxygenase (*LOX*), allene oxide cyclase (*AOC*), and fatty acid hydroperoxide lyase (*HPL*) were examined in the unripe green fruits of transgenic lines as well as green and red fruits of non-transformed pepper as controls. Results showed that three JA-related genes were highly expressed in the green fruits of transgenic lines (Figure 6A). The *LOX* gene was expressed more abundantly in the green fruits of transgenic lines, while the expression levels of *AOC* and *HPL* genes were approximately similar to those in the ripe fruits of non-transformed pepper. Since it has been known that disease resistance is related to the expression of defense-related genes such as PR genes, we further investigated the expression of two PR genes: methyl jasmonate (MeJA)-treatment induced *CaPR*10 gene [42] and SA-induced *PepThi* gene [28]. The results showed that both genes were expressed in the unripe green fruits of transgenic lines, whereas no expression was observed in the green fruits of non-transformed pepper (Figure 6B). In the transgenic pepper fruits, the expression levels of MeJA-inducible *CaPR*10 were higher than those in the red fruits of non-transformed pepper. In contrast, the expression levels of SA-inducible *PepThi* were lower than those in the red fruits of non-transformed pepper. These results suggest that overexpression of J1-1 in the unripe fruits induced PR genes, which might be responsible for the disease resistance of the transgenic peppers. Moreover, the results also suggest that J1-1 proteins induced in the transgenic plants were affected by both JA and SA signalings, in which JA is more important than SA.

### Fungal Resistance of the Transgenic Pepper Plants against *C. gloeosporioides*

**Figure 7:**
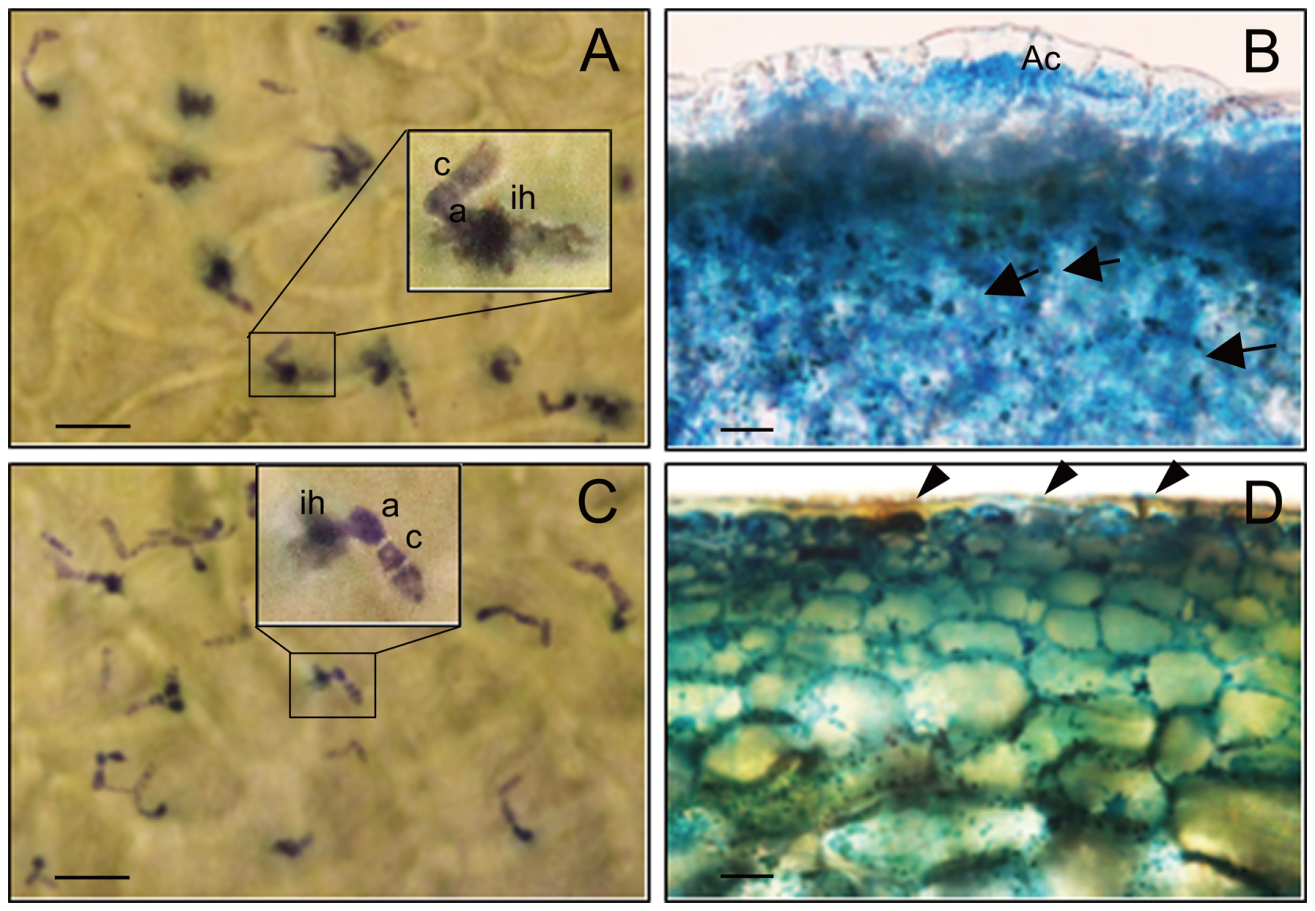
Inhibition of fungal growth in transgenic pepper fruits. **A & C,** Microscopic observation of fungal penetration at the infected area in the non-transformed (A) or J15 transgenic (C) unripe pepper fruit at 24 hr after inoculation with *C. gloeosporioides*. Fungus was stained with 0.1% toluidine blue. **B & D,** Cross sections of infection sites in the non-transformed (B) and J15 transgenic (D) fruits at 5 day after inoculation. Lactophenol-trypan blue was used for staining. a, appressorium; ih, infection hypha; c, conidium; Ac, acervuli. Arrowheads indicate spores and arrows indicate mycelia. Bar  = 25 µm.

To assess the efficacy of J1-1 protein against the anthracnose fungus, *in vivo* assay was conducted using the unripe fruits of four transgenic pepper lines. Spores were inoculated directly on the surface of detached green fruits and then observed for lesion development and spore formation. Within 24 hours, germinated conidium developed an appressorium and then penetrated into the cuticle layers in the unripe fruits of non-transformed wild-type pepper (Figure 7A). Prominent penetration marks were shown in the surroundings of the infection hypha on the outer surfaces and extensive fungal growth was observed in the lumen of fruit cells, which resulted in maceration and cell death at 5 days after infection (Figure 7B). On the contrary, the early infection process was compromised in the transgenic fruit, representing reduced cuticle penetration (Figure 7C). Moreover, the fungus was unable to colonize further in the transgenic pepper cells (Figure 7D).

**Figure 8:**
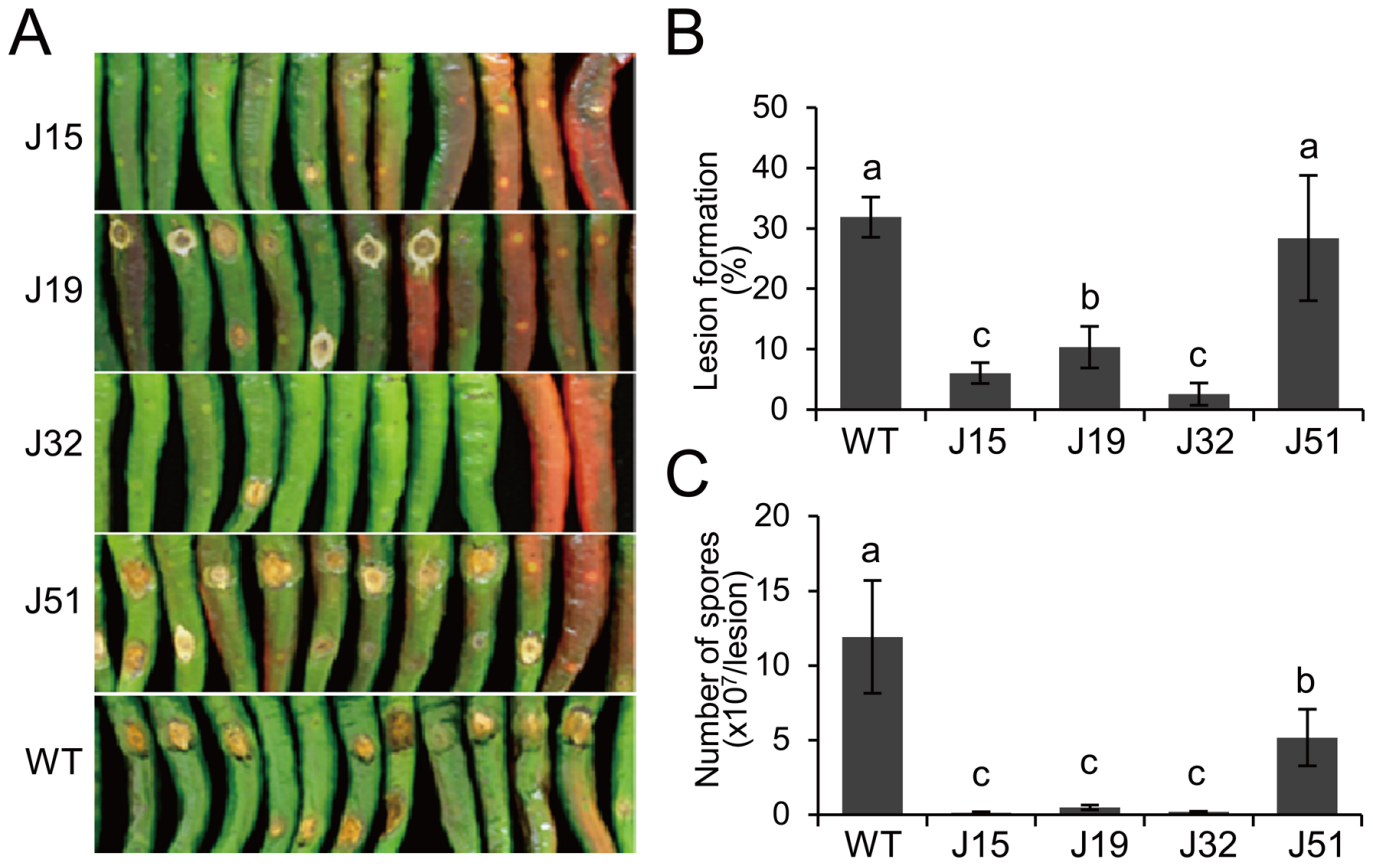
Fungal resistance of transgenic pepper fruits challenged with *C. gloeosporioides*. **A,** Representative photographs of unripe pepper fruits 9 days after infection with the anthracnose fungus. Green mature fruits from transgenic lines and wild-type control plants were inoculated with spores. J15, J19, J32 and J51, homozygous T_2_ transgenic pepper lines; WT, non-transformed unripe fruits as a negative control. **B,** The rate of lesion development from inoculated spots on infected fruits. **C,** Number of spores in a lesion of the infected fruits. Fifty unripe mature fruits were infected at two spots. The number of spores was counted in the infected area at 9 days after infection. The data are presented as means ± SD from three independent estimations. Means with different letters in each column are significantly different at P<0.05.

Nine days after inoculation, non-transformed wild-type fruits showed typical sunken disease symptoms of which spreading lesions were covered with soaked spores (Figure 8A). In contrast, transgenic fruits revealed very low frequency of lesion formation compared to the non-transgenic fruits (Figure 8B). Interestingly, necrotic lesions were hardly observed on the unripe fruit of J15 and J32 transgenic lines. In the case of the J19 and J51 lines, inoculated fruits tend to develop intermediate sized lesions with arid surface, implying limited spore formation. Thus, spore production was measured in the lesion to verify whether symptom restriction in transgenic plants was caused by inhibited fungal colonization (Figure 8C). After 9 days of incubation, the number of spores in all transgenic pepper lines was drastically lower than that of control plants, in which the J15 and J32 transgenic plants showed lower spore formation than other lines. This observation is consistent with the size of lesion on the inoculated unripe fruits. The J51 transgenic plants showing less restricted lesion development showed reduction by half in spore formation compared with the wild-type plant. Consequently, a strong correlation was observed between J1-1 protein and fungal resistance in the transgenic plants. These results confirmed that the unripe fruits accumulating a high level of J1-1 protein showed elevated resistance, indicating that lesion and spore developments were retarded by the action of J1-1 protein. Taken together, the results suggest that overexpression of *J1-1* in pepper plants leads to the restriction of fungal colonization by inhibiting fungal growth and spore production, and demonstrate that the J1-1 protein has a protective activity that prevents the spread of anthracnose symptoms in unripe pepper fruits.

## Discussion

This study finds new evidence that defensin is associated with a physiological process during phytopathogen interaction (Figure 1). Immunohistochemical study showed that massive J1-1 protein occurred in epidermal cells invaded by fungus. A noticeable amount of the peptide was found over the cell surface and surrounding the invading fungal conidium. Serial observation revealed that the peptide was excreted to the outside of the fruit. In addition, the recombinant J1-1/GST fusion protein showed inhibitory activity on the growth and development of the anthracnose fungus (Figure 2). This implies that the pepper defensin, J1-1, retains its biological activity similar to other defensins that exhibit antifungal effects [43]. Consequently, the initial contact of J1-1 with fungus can restrict fungal growth so that lesion formation might be effectively arrested during the early infection process.

Immunoblot analyses of J1-1 in various pepper tissues showed the expression of the protein in the ripe red fruit (Figure 1A). In addition, there was another defensin band with a higher size in flowers. This defensin is different from J1-1, because we could not detect the transcription of J1-1 in flowers (Figure S1). These results suggest the presence of two defensins in pepper, which is consistent with a previous report [27]. It has been shown that two defensin genes exist in the pepper genome, J1-1 (Gene accession no. X95363) and J1-2 (Gene accession no. X95730). Therefore, the defensin band in flowers is suspected as the other defensin gene (J1-2). Further studies will be necessary to elucidate the functional differences between flower-specific and fruit-specific defensins.

The present results showed that the production of J1-1 protein is regulated by ripening stages (Figure 1B and S2). To understand ripening related expression of J1-1, we retrieved promoter sequence of *J1-1* and analyzed for binding sites for transcription factors using PlantPAN (http://plantpan.mbc.nctu.edu.tw). The promoter contains the sequence motives related to known target sites for multiple consensus sequences for AtMYC2 and an ethylene responsive element (ERELEE4). These sequence motives are known to be involved in JA and ethylene signaling, respectively. Considering that pepper is known as non-climacteric fruit, this is consistent with our previous report that the expression of *J1-1* gene is up-regulated in the unripe pepper fruits by exogenous treatment of mehyl jasmonate [28].

During the T-DNA integration process, a short stretch of DNA can be deleted at the ends of the T-DNA or at the integration site of plant gDNA [44], [45]. To investigate whether such events have a special feature in pepper transformation, we cloned the flanking regions and examined the nucleotide sequences in the transgenic pepper lines that carry a single copy of T-DNA. In agreement with previous reports, deletions at the RB and LB of the T-DNAs were observed in pepper transgenic plants (Table 1 and S3). In our case, the length of deleted DNA varied according to the transgenic event, and a more extensive deletion of border sequences was observed, especially in the LB of T-DNA. The results might be related to the transformation efficiency being extremely low in pepper. Pepper is known to be a very recalcitrant plant to transform, with transformation efficiency reported to be as low as 0.05%–0.6% [38], [46]. According to the sequence of the T-DNA/gDNA junctions, improper maintenance of T-DNA significantly occurred in both the LB and RB in transgenic pepper plants. In the worst case, 28 bp was lost at the 3′ end of the *HPT* gene in the T-DNA integrated in J15. This may explain why the transformation of pepper is so difficult when using the *Agrobacterium*-mediated method. Severe loss of the border sequence of T-DNA may disturb the stable transformation process or the selection procedures during pepper transformation.

Once transgenic plants are established, the transgenes should be stably expressed in the plant over generations. To minimize genetic variation caused by the positional effect of T-DNA integration and complex transgene structure, it is necessary to screen genetically stable transformants to guarantee transgene inheritance in their progenies. However, the level and pattern of transgene expression may differ widely among transgenic pepper plants. In the present study, the mRNA level of the transgene was invariable among transgenic plants, but the accumulation of J1-1 protein was compromised in each transgenic line (Figure 5). According to the protein gel blot analysis, J1-1 protein was not maintained constantly in the unripe fruits during phytopathogen interaction (Figure S5). Consequently, a correlation between protein instability and the developmental state of fruits was observed for the J1-1 protein, indicating that the protein was seemingly not durable in the unripe fruit. Unlike other lines, the J51 line did not accumulate the J1-1 protein in healthy unripe fruit and showed transient accumulation of the transgene product after fungal infection (Figure 5B and S5B). This was unexpected because defensins are known to have inherent stability arising from the characteristic structure known as the CS*αβ* motif [19]. Pepper J1-1 protein was also relatively stable under high-temperature heating at 90°C for 10 min, representing 75% of antifungal activity (Figure 2). This discrepancy in protein stability between *in vivo* and *in vitro* might arise from the post-translational modification that is involved in the processing of J1-1 protein in a developmentally regulated manner.

The present study demonstrates that the J1-1 protein provided effective resistance of pepper fruits against fruit-specific anthracnose fungus (Figure 8). Symptom development in J15 and J32 compared to that in non-transformed control fruit revealed a crucial delay in the onset of the disease. By contrast, J51, with lower J1-1 accumulation, displayed similar lesion formation to the non-transformed control fruit, but reduced sporulation (Figure 8B and 8C). This might be explained that the J1-1 protein was detected in the J51 transgenic pepper fruits after fungal infection, although the level was significantly reduced (Figure S5). Considering that the J1-1 protein was not detected in the non-transformed control fruits, the J1-1 protein level induced in the J51 transgenic pepper fruits might be effective to reduce sporulation but not enough to reduce lesion formation. Collectively, our results indicate the significance of the J1-1 protein during phytopathogen interaction.

During the symptom development on the green pepper fruits by infection with the anthracnose fungus, the peripheral regions of infection sites tended to turn red in transgenic fruits (Figure 8A). This might be explained by the increased expression of JA-biosynthetic genes in the transgenic fruits (Figure 6A). Previously, exogenous JA treatment was shown to accelerate chlorophyll degradation but β-carotene accumulation in tomato [47], and endogenous level of JA was reported to be risen coincidently with the onset of ripening in apple and tomato fruit [41]. Therefore, elevated JA synthesis from the induction of JA-biosynthetic genes by J1-1 overexpression might account for green-to-red color change in the fruits after infection.

The present results also suggest that up-regulation of JA-biosynthetic genes in the unripe transgenic fruits might induce the expression of a defense-related gene such as the *CaPR*10 (Figure 6B). In addition, *C. gloeosporioides* is a hemibiotroph that start out as biotroph, but switched to necrotroph. SA-dependent responses are typically associated with resistance to biotrophs, whereas JA and ethylene synergistically regulate defense against necrotrophs. Thus, it is likely that, in association with the expression of other defense-related proteins, the constitutive expression of J1-1 resulted in sustainable tolerance levels of the transgenic plants to the fungus. However, further studies will be necessary to determine how overexpression of J1-1 protein contributes to the up-regulation of JA-biosynthetic genes. Conclusively, a pepper defensin, J1-1, exhibiting antimicrobial activities are quite versatile for biotechnological purposes to provide biological protection to pepper fruits.

## Supporting Information

Figure S1 **The expresseion of J1-1 gene in various organs of** ***C. annuum*** **.** The transcript levels were analyzed in leaf (Le), flower (Fl), unripe fruit (UF), and ripe fruit (RF) of *C. annuum* by RT-PCR. rRNA was shown as a loading control.
(PDF)

Figure S2 **Developmentally regulated J1-1 production in pepper fruits during ripening at stages I through V.** Stage I, green fruit; stage II, early breaker fruit; stage III, turning fruit: stage IV, purple fruit; stage V, red fruit. Total soluble proteins from the fruit were subjected to SDS-PAGE, blotted onto a PVDF membrane, and incubated with polyclonal J1-1 antibody. β-tubulin was shown as a loading control.
(PDF)

Figure S3 **J1-1 recombinant protein affect the development of** ***C. gloeosporioides*** **, in vitro.** **A** Appressorium formation. **B** Spore germination. Spore suspensions were amended with 10 µL of the GST/J1-1 recombinant protein or heated protein to final concentrations of 0.001, 0.01, 0.1, and 1 mg·mL^−1^. The protein was heated by incubating at 90°C for 10 min. A minimum of 100 spores were counted per replicate. Each value represents the mean ± SD of three replicates. Means with different letters in each column are significantly different at P<0.05.
(PDF)

Figure S4 **Development of transgenic plants from pepper explants. A** Pepper seeds were germinated in the dark and incubated on a half strength MS medium for 6 days. **B** Hypocotyl and cotyledonary explants were pre-incubated on callus induction medium for two days. **C** After *Agrobacteria* infection, the explants were incubated in the callus induction medium containing 20 mg·L^−1^ hygromycin and 400 mg·L^−1^ cefotaxime. **D** Callus was incubated on the shoot induction media containing 10 mg·L^−1^ hygromycin. **E** The regenerated shoots were transferred onto a root inducing media. **F** Regenerated putative transgenic plant.
(PDF)

Figure S5 **Expression of the** ***J1-1*** **in infected unripe pepper fruits. A** Northern blot analysis. Unripe fruits from transgenic and wild-type plants at 0 and 24 hours after inoculation (HAI) with *C. gloeosporioides* were used in this analysis. **B** Immunoblot analysis. Total soluble proteins from T_2_ progenies were subjected to immunoblot analysis with polyclonal anti-J1-1 antibody. WT, infected unripe fruits of wild type; J32 and J51, infected unripe fruits of respective transgenic plants. rRNA and β-tubulin were shown as loading controls.
(PDF)

Table S1 **Primers used in this study.**
(PDF)

Table S2 **Segregation ratios for hygromycin resistance in the progenies of transgenic peppers.**
(PDF)

Table S3 **Rescued sequences of T-DNA/gDNA junctions in the J15, J19 and J51 transgenic lines.** RB, Right border; LB, Left border. Plant genomic DNA sequence is indicated in gray. The primer sequences used for i-PCR are underlined, and the restriction sites are in italic.
(PDF)
