# Supplementary material for: Overexpression of a Defensin Enhances Resistance to a Fruit-Specific Anthracnose Fungus in Pepper
Source: PLoS One. 2014 May 21;9(5):e97936. doi: 10.1371/journal.pone.0097936 (PMC4029827; doi:10.1371/journal.pone.0097936)
Supplement: Figure S1 — The expresseion of J1-1 gene in various organs of C. annuum . The transcript levels were analyzed in leaf (Le), flower (Fl), unripe fruit (UF), and ripe fruit (RF) of C. annuum by RT-PCR. rRNA was shown as a loading control. (PDF) [file pone.0097936.s001.pdf]

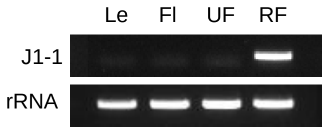

Figure S1. The expresseion of *J1-1* gene in various organs of *C. annuum*. The transcript levels were analyzed in leaf (Le), flower (Fl), unripe fruit (UF), and ripe fruit (RF) of *C. annuum* by RT-PCR. rRNA was shown as a loading control.
