# Supplementary material for: Overexpression of a Defensin Enhances Resistance to a Fruit-Specific Anthracnose Fungus in Pepper
Source: PLoS One. 2014 May 21;9(5):e97936. doi: 10.1371/journal.pone.0097936 (PMC4029827; doi:10.1371/journal.pone.0097936)
Supplement: Figure S2 — Developmentally regulated J1-1 production in pepper fruits during ripening at stages I through V. Stage I, green fruit; stage II, early breaker fruit; stage III, turning fruit: stage IV, purple fruit; stage V, red fruit. Total soluble proteins from the fruit were subjected to SDS-PAGE, blotted onto a PVDF membrane, and incubated with polyclonal J1-1 antibody. β-tubulin was shown as a loading control. (PDF) [file pone.0097936.s002.pdf]

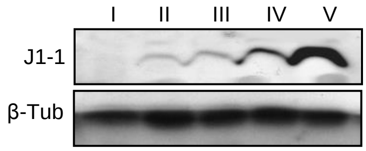


**Figure S2. Developmentally regulated J1-1 production in pepper fruits during ripening at stages I through V.** Stage I, green fruit; stage II, early breaker fruit; stage III, turning fruit: stage IV, purple fruit; stage V, red fruit. Total soluble proteins from the fruit were subjected to SDS-PAGE, blotted onto a PVDF membrane, and incubated with polyclonal J1-1 antibody. β-tubulin was shown as a loading control.
