# Supplementary material for: Overexpression of a Defensin Enhances Resistance to a Fruit-Specific Anthracnose Fungus in Pepper
Source: PLoS One. 2014 May 21;9(5):e97936. doi: 10.1371/journal.pone.0097936 (PMC4029827; doi:10.1371/journal.pone.0097936)
Supplement: Figure S3 — J1-1 recombinant protein affect the development of C. gloeosporioides , in vitro. A Appressorium formation. B Spore germination. Spore suspensions were amended with 10 µL of the GST/J1-1 recombinant protein or heated protein to final concentrations of 0.001, 0.01, 0.1, and 1 mg·mL−1. The protein was heated by incubating at 90°C for 10 min. A minimum of 100 spores were counted per replicate. Each value represents the mean ± SD of three replicates. Means with different letters in each column are significantly different at P<0.05. (PDF) [file pone.0097936.s003.pdf]

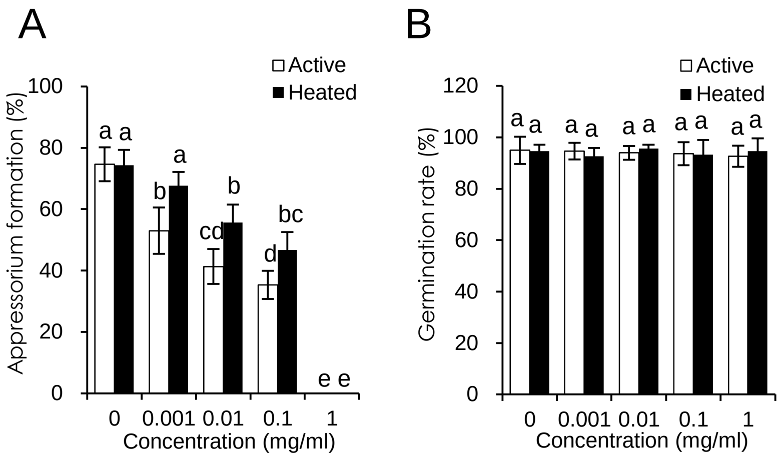

Figure S3. **J1-1 recombinant protein affect the development of *C. gloeosporioides*, *in vitro*.** **A** Appressorium formation. **B** spore germination. Spore suspensions were amended with 10 μL of the GST/J1-1 recombinant protein or heated protein to final concentrations of 0.001, 0.01, 0.1, and 1 mg/mL. The protein was inactivated by incubating at 90°C for 10 min. A minimum of 100 spores were counted per replicate. Each value represents the mean ± SD of three replicates. Means with different letters in each column are significantly different at $P < 0.05$.
