# Supplementary material for: Overexpression of a Defensin Enhances Resistance to a Fruit-Specific Anthracnose Fungus in Pepper
Source: PLoS One. 2014 May 21;9(5):e97936. doi: 10.1371/journal.pone.0097936 (PMC4029827; doi:10.1371/journal.pone.0097936)
Supplement: Figure S4 — Development of transgenic plants from pepper explants. A Pepper seeds were germinated in the dark and incubated on a half strength MS medium for 6 days. B Hypocotyl and cotyledonary explants were pre-incubated on callus induction medium for two days. C After Agrobacteria infection, the explants were incubated in the callus induction medium containing 20 mg·L−1 hygromycin and 400 mg·L−1 cefotaxime. D Callus was incubated on the shoot induction media containing 10 mg·L−1 hygromycin. E The regenerated shoots were transferred onto a root inducing media. F Regenerated putative transgenic plant. (PDF) [file pone.0097936.s004.pdf]

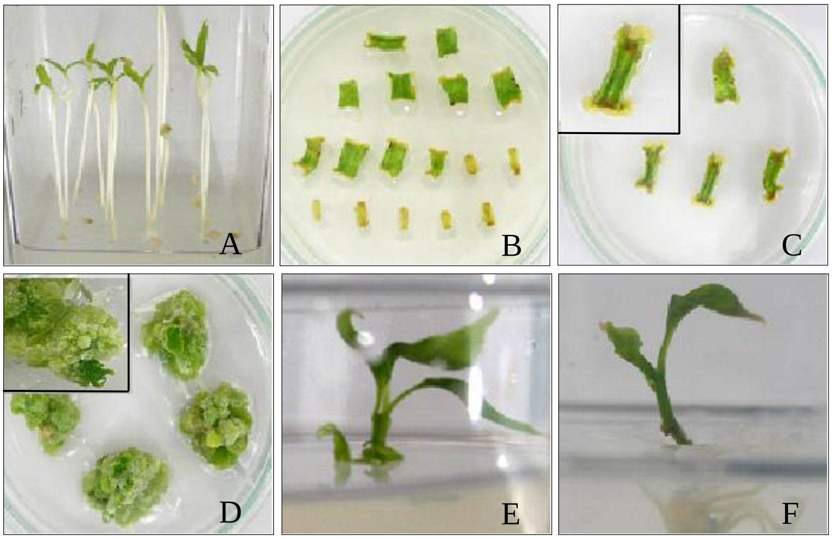

Figure S4. Development of transgenic plants from pepper explants. A Pepper seeds were germinated in the dark and incubated on a half strength MS medium for 6 days. **B** Hypocotyl and cotyledonary explants were pre-incubated on callus induction medium for two days. **C** After *Agrobacteria* infection, the explants were incubated in the callus induction medium containing 20 mg L^{-1} hygromycin and 400 mg L^{-1} cefotaxime. **D** Callus was incubated on the shoot induction media containing 10 mg L^{-1} hygromycin. **E** The regenerated shoots were transferred onto a root inducing media. **F** Regenerated putative transgenic plant.
