# Supplementary material for: Overexpression of a Defensin Enhances Resistance to a Fruit-Specific Anthracnose Fungus in Pepper
Source: PLoS One. 2014 May 21;9(5):e97936. doi: 10.1371/journal.pone.0097936 (PMC4029827; doi:10.1371/journal.pone.0097936)
Supplement: Figure S5 — Expression of the J1-1 in infected unripe pepper fruits. A Northern blot analysis. Unripe fruits from transgenic and wild-type plants at 0 and 24 hours after inoculation (HAI) with C. gloeosporioides were used in this analysis. B Immunoblot analysis. Total soluble proteins from T2 progenies were subjected to immunoblot analysis with polyclonal anti-J1-1 antibody. WT, infected unripe fruits of wild type; J32 and J51, infected unripe fruits of respective transgenic plants. rRNA and β-tubulin were shown as loading controls. (PDF) [file pone.0097936.s005.pdf]

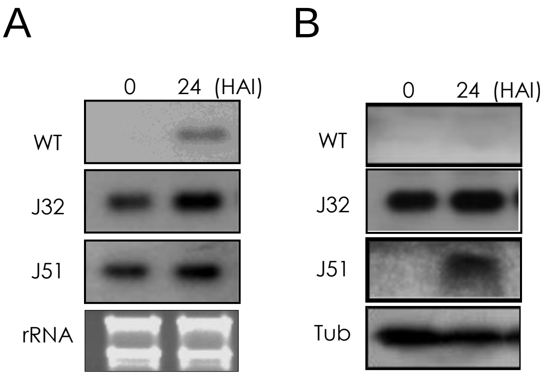

Figure S5. Expression of the *J1-1* in infected unripe pepper fruits. A Northern blot analysis. Unripe fruits from transgenic and wild-type plants at 0 and 24 hours after inoculation (HAI) with *C. gloeosporioides* were used in this analysis. **B** Immunoblot analysis. Total soluble proteins from T_2 progenies were subjected to immunoblot analysis with polyclonal anti-J1-1 antibody. WT, infected unripe fruits of wild type; J32 and J51, infected unripe fruits of respective transgenic plants. rRNA and β-tubulin were shown as loading controls.
