# Supplementary material for: Overexpression of a Defensin Enhances Resistance to a Fruit-Specific Anthracnose Fungus in Pepper
Source: PLoS One. 2014 May 21;9(5):e97936. doi: 10.1371/journal.pone.0097936 (PMC4029827; doi:10.1371/journal.pone.0097936)
Supplement: Table S1 — Primers used in this study. (PDF) [file pone.0097936.s006.pdf]

Table S1. Primers used in this study.

Primer code	Sequences	use
IP F1	5’-CCGATCGTTCAAACATTTGGCAATAAAGTT-3’	i-PCR
IP F2	5’-TCTTAAGATTGAATCCTGTTGCCGGTCTTG-3’	
IP F3	5’-GTGTAGAAGTACTCGCCGATAGTGGAAACC-3’	
IP F4	5’-CCATAATAATGTGTGAGTAGTTCCCAGATAA-3’	
IP R1	5’-CTGTGTTCTTGATGCAGTTAGTCCTGAATC-3’	
IP R2	5’-TTCCTGGAGATTATTGCTCGGGTAGATCGT-3’	
IP R3	5’-GCTCTAGCCAATACGCAAACCGCCTCT-3’	
IP R4	5’-CAGTGAGCGCAACGCAATTAATGTGAGT-3’	
LOX F	5’-CCGTATCCTCGTAGAGGCA-3’	Probe for Northern blot
LOX R	5’-CATCCGTCCTCCATGCAGT-3’	
AOC F	5’-CATGGCCACTGCTTCCTCA-3’	
AOC R	5’-CCGTAAGCACCGAAGTAGAAACT-3’	
CaHPL F	5’-GCGGTACTGGATGTCAAGTCATTTG-3’	
CaHPL R	5’-GAACCTGAAAAGTTTATGTTGGAGAGG-3’	
*CaPR*10 F	5’-GCACGGCAATCATCTTATCCTA-3’	
*CaPR*10 R	5’-GTTCTTTCCATGACAACCAATTG-3’	
*Pep*Thi F	5’-ATGGCTCGTTCCATTTACTTCATG-3’	
*Pep*Thi R	5’-TATTTAATTTTGTGTGACACT-3’	
J1-1 F	5’-ATGGCTGGCTTTTCCAAAGTAG-3’	RT-PCR
J1-1 R	5’-CACAGGGCTTCGTGCAGAAG-3’	
rRNA F	5’-CGGTAATTCCAGCTCCAATAGC-3’	
rRNA R	5’-CCATGCTAATGTATACAGAGCGTAGG-3’	
