# Supplementary material for: Overexpression of a Defensin Enhances Resistance to a Fruit-Specific Anthracnose Fungus in Pepper
Source: PLoS One. 2014 May 21;9(5):e97936. doi: 10.1371/journal.pone.0097936 (PMC4029827; doi:10.1371/journal.pone.0097936)
Supplement: Table S2 — Segregation ratios for hygromycin resistance in the progenies of transgenic peppers. (PDF) [file pone.0097936.s007.pdf]

Table S2. Segregation ratios for hygromycin resistance in the progenies of transgenic peppers.

Line	Generation	Germinated[a]	Resistant[b]	Sensitive[c]	χ^{2} [d]	Ratio[e]
15	T_1	47	33	14	0.56	3:1
15-1	T_2	47	47	0		(Homo)
15-4	T_2	46	46	0		(Homo)
19	T_1	43	34	9	0.37	3:1
19-2	T_2	48	48	0		(Homo)
19-7	T_2	32	32	0		(Homo)
32	T_1	48	35	13	0.11	3:1
32-2	T_2	48	48	0		(Homo)
32-3	T_2	40	40	0		(Homo)
51	T_1	48	35	13	0.11	3:1
51-4	T_2	46	46	0		(Homo)
51-6	T_2	48	48	0		(Homo)

[a]Number of germinated seeds on MS medium containing 20 mg L^{-1} hygromycin.
[b]Number of seedlings survived on the medium containing 20 mg L^{-1} hygromycin.
[c]Number of seedlings sensitive on the medium containing 20 mg L^{-1} hygromycin.
[d]Significantly different at $P < 0.05$.
[e]Ratio of resistant versus sensitive seedlings on hygromycin.
