# Supplementary material for: Overexpression of a Defensin Enhances Resistance to a Fruit-Specific Anthracnose Fungus in Pepper
Source: PLoS One. 2014 May 21;9(5):e97936. doi: 10.1371/journal.pone.0097936 (PMC4029827; doi:10.1371/journal.pone.0097936)
Supplement: Table S3 — Rescued sequences of T-DNA/gDNA junctions in the J15, J19 and J51 transgenic lines. RB, Right border; LB, Left border. Plant genomic DNA sequence is indicated in gray. The primer sequences used for i-PCR are underlined, and the restriction sites are in italic. (PDF) [file pone.0097936.s008.pdf]

Table S3. Rescued sequences of T-DNA/gDNA junctions in the J15, J19 and J51 transgenic lines. RB, Right border; LB, Left border. Plant genomic DNA sequence is indicated in gray. The primer sequences used for i-PCR are underlined, and the restriction sites are in italic.

Line		DNA sequence	
J15	RB	F1 Primer	
		**CCGATCGTTCAAACATTTGGCAATAAAGTTTCTTAAGATTGAATCCTGTTGCCGGTCTTG**	60
		**CGATGATTATCATATAATTTCTGTTGAATTACGTTAAGCATGTAATAATTAACATGTAAT**	120
		**GCATGACGTTATTTATGAGATGGGTTTTTATGATTAGAGTCCCGCAATTATACATTTAAT**	180
		**ACGCGATAGAAAACAAAATATAGCGCGCAAACTAGGATAAATTATCGCGCGCGGTGTCAT**	240
		**CTATGTTACTAGATCGG*GAATTC***CTTACCACACCATTGTGCATTCTAATTTTGACTATAC	300
		CTTTACCAAAAAAATTTCAAGGAGCATTGTTGCTTATCAAGACAACTCCACCTCCAACAT	360
		ATATGAATGTGGTAAAAAAATACCTATAGGGACACATATGATAAGAACAACCTAAATTCA	420
		AAATGCACTCATCGTTAGATTTAAAACTATTGTTAGTTGCTAAAAATATTGTTCGCTCAG	480
		ACTCATAAGCAACTACACTTGATTTGGCAGAGTCAGCATTTTGGTTCTTATTTTTGTTTT	540
		CCTTGTGCTTTTCTTTTTTATTCAACTTATAGCATTTAGAAATAACACGACCTTTCTTAT	600
		GACAGCATTTGCACACTACATATCTGTATTTATACTTTGACCTTGATTTATACCGCTCAC	660
		TATTTGGTTCTTTCTTATTAGATCTACCTCTTACAAATAATCCTTCCCCACGAGTTGCGC	720
		TGCTTTCCCGAGTAATATCTCTATCTATCTGATTCTTTGTTTTTAAGATAGATTTGATTT	780
		CTCTATAAGAGATATTATCCTTTCCATAAAGTATAGTATCTCTAATATGCTTAAATGATT	840
		AGGGTAAGAAAATAATCAATAAGACGGATTAATCTTCATCATTAACTTCAAAGCCTATAT	900
		TACTTAAGAATAGAATCAAAAGCATGAAGATATGAGATTATAGAAGTATCTCCAACCATA	960
		CAAAATGTGTAAAGCTTTTGCTTCAGGTAAAGTCTATTTTCCACTCTCCTTTTCATATAC	1020
		AAGGTTTTTAGTTTTTCCCGCATTTCTTTCATTGTGGTTTCTGCAGATACTTCATGTAAA	1080
		ACCTCATTTGAGAGATTTAGAATGGTACCTAATTTTGCCTTTTTGTATATGACGGCAAAC	1140
		TCTTCATCTGTAATATTTTCTGGCTTCATCTCCTTTCCTTCCAATGTCATATCTAAGCCA	1200
		TCCTGAATTAAGATAGCTTGCATCTTTAATTGCCACATCCCAAAGTTTGCACCTCGATCA	1260
		AAATTTTCAACGTAAATCTTTGTTATTTTATTTTGGCTGCTGAAATAACATGGTTAGACC	1320
		CGACTCTGATACCAATTTGTTAGGATCGAGAACCCAATTATTGCGAAATTTAGCCAAACA	1380
		TGCTTTAAGGAAAATAACAAAGACAGTAACAAGTTGACAATAATGGTAATTAATAAAATA	1440
		AAGGAGGCACAAATTTAATGTGATTCGGTCATGTGACCTACGTCCACGATCAATTTCACC	1500
		ATATCAACAAGAGTACAATATTGTGGTTACAAAATTAGAGTAGTCACTCTAATTAACCCA	1560
		AATATTCCAAGAGAACAACCTCTGAAGATTACTCCAAAAGAAGGGCTCACACAAGTGTTT	1620
		CCCAACACCCACTCTCTAACTAAATATTCTAATTAAAAGGGAGAAGAAATATAAGAGACT	1680
		GAAAGAGAACTCTTGAAATTGATATGTTATGAATGAGAAAGTAGGTCCCTATTTATAGAG	1740
		ATGAAAATGGGCTTGTTGATGTTATCCATGATGTTAATGGAAGATGTAAATTTCAACATT	1800
		TGCATGTGAATGAGAGAAATTTACAACAAAATCTTTACTAGCAACTTTGTTTGACAAAAC	1860
		CAACAAATAATCTTCCAAAAAGTCTTTGGTTTGCTTTTACCCAGTGTGAACCAAAGAAAA	1920
		AACAAAGGCCATATTATATTAACCATTCAAACTACAAAGGATATTGCTCAAATAATCTTA	1980
		GATGTCCTTTATGTTCTAGAGTTGACGTCAAATCTTGAGTCTTGGTCAGTTGCAAGAGAA	2040
		AGGATATGAGATTATCATTAAAAATAGATTTTGTCGATAATAATTTGAGATAATTGCTCA	2100
		AGCTAAAATGACTGCAAACAACAGAATGGTTCCTCTCTATCTCAATAATATTCAACAATC	2160
		ATACTTCTTAGAAAAACAAGGAGATGGTGCCTTGTTGTGGCATTTTCGACAACAACCAGA	2220
		TAAGG**GATAGTTTAAACTGAAGGCGGGAAACGACAATCTGATCCAAGCTCAAGCTGCTCT**	2280
		**AGCATTCGCCATTCAGGCTGCGCAACTGTTGGGAAGGGCGATCGGTGCGGGCCTCTTCGC**	2340
		**TATTACGCCAGCTGGCGAAAGGGGGATGTGCTGCAAGGCGATTAAGTTGGGTAACGCCAG**	2400
		**GGTTTTCCCAGTCACGACGTTGTAAAACGACGGCCAGTGCCAAGCTTGCATGCCTGCAGG**	2460
		**TCCCCAGATTAGCCTTTTCAATTTCAGAAAGAATGCTAACCCACAGATGGTTAGAGAGGC**	2520
		**TTACGCAGCAGGTCTCATCAAGACGATCTACCCGAGCAATAATCTCCAGGAAATCAAATA**	2580
		**CCTTCCCAAGAAGGTTAAAGATGCAGTCAAAAGATTCAGGACTAACTGCATCAAGAACAC**	2640
		**AG** R1 primer	2642
	LB	F3 primer	
		**GAAGTACTCGCCGATAGTGGAAACCGAAGACCA**TTCATGTTGTTGCTCAGGTCGCAGACG	60
		TTTTGCAGCAGCAGTCGCTTCACGTTCGCTCGCGTATCGGTGATTCATTCTGCTAACCAG	120
		TAAGGCAACCCCGCCAGCCTAGCCGGGTCCTCAACGACAGGAGCACGATCATGCGCACCC	180
		GTGGCCAGGACCCAACGCTGCCCGAGATGCGCCGCGTGCGGCTGCTGGAGATGGCGGACG	240
		CGATGGATATGCTCTGCCAAGGGTTGGTTTGCGCATTCACAGTTCTCCGCAAGAATTGAT	300
		TGGCTCCAATTCTTGGAGTGGTGAATCCGTTAGCGAGGTGCCGCCGGCTTCCATTCAGGT	360
		CGAGGTGGCCCGGCTCCATGCACCGCGACGCAACGCGGGGAGGCAGACAAGGTATAGGGC	420
		GGCGCCTACAATCCATGCCAACCCGTTCCATGTGCTCGCCGAGGCGGCATAAATCGCCGT	480
		GACGATCAGCGGTCCAATGATCGAAGTTAGGCTGGTAAGAGCCGCGAGCGATCCTTGAAG	540
		CTGTCCCTGATGGTCGTCATCTACCTGCCTGGACAGCATGGCCTGCAACGCGGGCATCCC	600
		GATGTCGCCGGAAGCGAGAAGAATCATAATGGGGAAGGCCATCCAGCCTCGCGTCGCGAA	660
		CGCCAGCAAGACGTAGCCCAGCGCGTCGGCCGCCATGCCGGCGATAATGGCCTGCTTCTC	720
		GTCGAAACGTTTGGTGGCGGGACCAGTGACGAAGGCTTGAGCGAGGGCGTGCAAGATTCC	780
		GAATACCGCAAGCGACAGGCCGATCATCGTCGCGCTCCAGCGAAAGCGGTCCTCGCCGAA	840
		AATGACCCAGAGCGCTGCCGGCACCTGTCCTACGAGTTGCATGATAAAGAAGACAGTCAT	900
		AAGTGCGGCGACGATAGTCATGCCCCGCGCCCACCGGAAGGAGCTGACTGGGTTGAAGGC	960
		TCTCAAGGGCATCGGTCG**AGATCTCGGTGCCTAATGAGTGAGCTAACTTACATTAATTGC**	1020
		**GTTGCGCTCACTGCCCGCTTTCCAGTCGGGAAACCTGTCGTGCCAGCTGCATTAATGAAT**	1080
		**CGGCCAACGCGCGGGGAGAGGCGGTTTGCGTATTGGCTAGAG**	1140
		R3 primer	

**J19**	RB	F1 primer<br>**CCGATCGTTCAAACATTTGGCAATAAAGTTTCTTAAGATTGAATCCTGTTGCCGGTCTTG** 60<br>**CGATGATTATCATATAATTTCTGTTGAATTACGTTAAGCATGTAATAATTAACATGTAAT** 120<br>**GCATGACGTTATTTATGAGATGGGTTTTTATGATTAGAGTCCCGCAATTATACATTTAAT** 180<br>**ACGCGATAGAAAACAAAATATAGCGCGCAAACTAGGATAAATTATCGCGCGCGGTGTCAT** 240<br>**CTATGTTACTAGATCGG*GAATTC***GGGCCGTAAATTGGATTAAAAATGCTCAATTTTTCAA 300<br>ACTTAAAGGGCCATTACGCGCCCAGAGTAAGACAACAATGATAAAATTAGACTCACTGTA 360<br>AATT**TGAAGGCGGGAAACGACAATCTGATCCAAGCTCAAGCTGCTCTAGCATTCGCCATT** 420<br>**CAGGCTGCGCAACTGTTGGGAAGGGCGATCGGTGCGGGCCTCTTCGCTATTACGCCAGCT** 480<br>**GGCGAAAGGGGGATGTGCTGCAAGGCGATTAAGTTGGGTAACGCCAGGGTTTTCCCAGTC** 540<br>**ACGACGTTGTAAAACGACGGCCAGTGCCAAGCTTGCATGCCTGCAGGTCCCCAGATTAGC** 600<br>**CTTTTCAATTTCAGAAAGAATGCTAACCCACAGATGGTTAGAGAGGCTTACGCAGCAGGT** 660<br>**CTCATCAAGACGATCTACCCGAGCAATAATCTCCAGGAAATCAAATACCTTCCCAAGAAG** 720<br>**GTTAAAGATGCAGTCAAAAGATTCAGGACTAACTGCATCAAGAACACAGA** 757<br>R1 primer
	LB	F3 primer<br>**GTGTAGAAGTACTCGCCGATAGTGGAAACCGACGCCCCAGCACTCGTCCGAGGGCAAAGA** 60<br>**AATAGAGTAGATGCCGACCGGATCTGTCGAT**TTTTTGGGTACAAGTAACCTGTCGGTCAA 120<br>AGTCATTGCTTTTCCTAGGAGTATGACATGGATTAGTATTCAAACATTGGCTCAAGACGT 180<br>GCCTTATGTGGGACAATATTCAACTTAGGAACTAAGGGGTTGTTTTGATACGTAGACTAA 240<br>ATTATCTCGGTATTATAATCTTGGGATTATAATTCTTGGACTATTTTGTACGACTAGTCC 300<br>CATGGTTCATGGGATAAGATGGGTTATACCGAGACTAAATTTAGTATTAGCTTTATACCA 360<br>TGTTTGGTCGATGGTGTAAATTAGTACCGGGATAAATATACCATCAACCAAATATGGAAT 420<br>AAATATTATCCATGGGATATATCAAACTTATATCTCCTACCAGACGACCCCTAAGTACTA 480<br>ACAATGCTAACATCCTAACACCAGGGAAATGAAATAGTGCCATTGTTGGTTCATAATGAA 540<br>TGAGGTTCTTCTGGAGATTTGTATTCTCTTGTGGATTTCATTTGACCATTTCTTCCATCC 600<br>TACTCGAACCAGTGACATATCTGTAGCTATTGCCTGCAATGTAATGTCATAAAACTCTTT 660<br>CTCTTTTACTTCATCTGTATAATTGCTAGAACTGTAACCAAAACTATATTTTACTAGTAG 720<br>TCAAGCTACCACCAATAATCAGTTATTTTTCTTTTATAGTACACATATTTATTTTCCTGC 780<br>TCTTGGGATTCTGTCTCCTACATGTTGACGTCTCTGTTATCACAAACAATCATACACATT 840<br>TTCATGTTTTCATGTTCCAAAATCTACTTTAATGATGGAGATAGATCTAATCATTTGAAT 900<br>TGCCTTTTCATTCCTCTCTCCCTTCACCCACATTATCAATCTGCTTTCTCTATTCTACTT 960<br>ACTGATCTTCTACAACTTTGATATAACTTCTCTATCTATCCCGATCTAACATAGAATTGC 1020<br>CTTCATAAATACTTAAGGATCAACCTAGCTAGGTTTGTCCCTTGTCTACTGTCAAGATTT 1080<br>TCATATCTAGTTTAGAACTTTTTAATCATTTATCCAAGCAACGATCTTGGGAATCGAGAC 1140<br>ATAGAGAACTTTTTAATCATTTACAAAATAATTGATGAATAATTTCCATCTGTTGTTACA 1200<br>AAATAATAGTTTCCTCTCTTAATGAGTCTACTATCTATTTCTAAGTGGTAGTGTTGAGAT 1260<br>ACAGAGACAAAAGAAAATGAGAGAAACCTGTGCATTCTCTCATGGAATTTCGATTTACTT 1320<br>TTTACTGCAACATTACTTTACCCTGATTTACTAGTTGGTGTGGATATTACGTGAATACTT 1380<br>CATAATGATTCTCTAAATTTCTTCGGTGTATCATTAGTATGGAGAATATGCTCAGTACTC 1440<br>TAGTTAGGAAACAGACTTCTATTGGACCATTTCTTTTAAAAAATAATTTGTTGATACTTC 1500<br>AAGAAAATCTTACAGGGAAGCATTGGAGTAACTGTAAAGTTGCTTCCATGTGACCAAGAG 1560<br>GTCGGCATACAAGTTGTGAAGAAATAGAACATCTATACAATAACACTCTCTGA***AAGCTT*G** 1620<br>**CATGCCTGCAGGTCCCCAGATTAGCCTTTTCAATTTCAGAAAGAATGCTAACCCACAGAT** 1680<br>**GGTTAGAGAGGCTTACGCAGCAGGTCTCATCAAGACGATCTACCCGAGCAATAATCTCCA** 1740<br>**GGAAAA** R2 primer 1746
**J51**	LB	F3 primer<br>**GTGTAGAAGTACTCGCCGATAGTGGAAACCGACGCCCCAGCACTCGTCCGAGGGCAAAGA** 60<br>**AATAGAGTAGATGCCGACCGGGATCTGTCGATCGACAAGCTCGAGTTTCTCCATAATAAT** 120<br>**GTGTGAGTAGTTCCCAGATAAGGGAATTAGGGTTCCTATAGGGTTTCGCTCATGTGTTGA** 180<br>**GCATATAAGAAACCCTTAGTATGTATTTGTATTTGTAAAATACTTCTATCAATAAAATTT** 240<br>**CTAATTCCTAAAACCAAAATCCAGTACTAAAATCCAGATCCCCCGAATTAATTCGGCGTT** 300<br>**AATTCAGTACATTAAAAACGTCC**ACAAAGTTTGGAACCATGCACTATCCTTCTTGTGGAT 360<br>AAGCATCCTCACAATCATCATCATCATCATCATTATGTGGCTCATTTTTGGGTTCCTCTC 420<br>CATCTCGGGGCTCAGCCTCATTCTCTTCTTTTTCAAAACCCACTTCTTCACCAAGGTAAT 480<br>ACAATATTTCTTCCCTTAGAATCACATTGCGTCGGTTTGGACATTCATTGGTTTTATGTC 540<br>CCCAATCTTGGCACTTGAAATATTAGAACCCTTTAGGATTATGATACTTGTGAGCTTCTT 600<br>GCCGGGAAGGAAATTTGTGGACTATTTTAGGCTCGGGCAGCCTTATAGTGGCGGGTTGGT 660<br>CCTTGTTTTTGGGCCAACCCGAAGATGGTTGAACCATATTTTTGCTCCTCAACCAACCCG 720<br>AGGTTGATGGTCCCTTTTGCCTGGACGATGACCTCTCTTTAAGCTCCCTTTCAATCTCAA 780<br>GAGCCGCTTGGAAAATTCCTTCACGGTATTCAACTTGTGAAGTGTCAAATGAGTGGAGAT 840<br>CTCTTCGTTCCACCCACACTTGAATCGAATGATGTCATGGCCAATTTGTTCTTCACGATG 900<br>ATCAAGCTTCAACATGAGTTGTTGAAACTCATCATAATATGCCATCACACTTTGATTCCC 960<br>TTTTCGCAAATTGTACAACCTAGCAAGTAACTCATGACGATAACTTTTGGGAAGATATCG 1020<br>TTGCCTCATTAGATACCTCAACCAAAACCATGGAGGTAGTTTTCCATCAATTCATTCATT 1080<br>ACCAAACCGTTTGACATATTCCTACCATGTGTTAGCATAGCCTTCAAAGTGAGCTATGGC 1140<br>ATAACAACTCTTCTTCTCCTCCGATATATCATTCACTTGAAATACTTTATCACACGCCGA 1200<br>CTCCTAAGCAAGATACACCTCGGGGTCACTTTCACCCTTAAAGATAGGTAGGCTTAATTT 1260<br>GATGGTATTGATGCCTACGTCTCTCTCTTGGTATCGGTTTACCCTTTCTCCATACCCTCG 1320<br>GTATATTCTTGGATCCACACATTCCCCTCGCACCTCCTTTTCGCTCATGTAAGCATCGTT 1380<br>AAAGGCTCCATACCCTTCATAATACTCTCTATCATATTCCTCAAAGTGGACGAGATGGTT 1440<br>TTGGACGTTTAGAACTTGATTTAGAGGATTGGATTTTGCGGAGGGGGTGGTCTTTGGTTT 1500<br>AGTGGAGCTTGGAAGGGAGGTTTCGGATTTTGTGGAGGTATTGACGTCCAAGTCCCTCCT 1560<br>GCAGAACTTGAAGAGTTTGGAAATGGGTTGGTCTAGTGGAGTCTCGATTTAGGGGGTAAG 1620<br>GAGTGGTTTGGCTTGCGGCATTTAGTGGAGCTAGTGAAGGATTTGGCAACTGTGGCCATG 1680

		TTTCGGGAGTAACGGTGGTGGAGGAATTTCTATCATGTCCACTTGACGAAACATGTTGAG 1740<br>GAGCAGAAGGATGAGAGTTCCTTTGACTCTCCACTTGTTCTAACCTCCCACTAATAGTCG 1800<br>TCACTTCTCCCCTTATGGCTCTCACTTCCATACTCAACCTTTCAAGAGCTCGGGCATAGC 1860<br>CTCAAGAGTGACCTCATATTCTCCATTTTATTAGAATCCACGATTTGAGACGTGGTTCCC 1920<br>TCAATTGGCAAGGTATTACCTATACAAGCAACAAACAAGTTAGTTTAAAACCTCTCCACA 1980<br>CTCACAGACTCTCGGATCACTCACACTCGAGCTCTACAAGTGTTGTAGATTGTCTAGTAA 2040<br>CCCAT***GAATCC*****TTAAGGTTTGAGTATGCTCTTGTCCAGAGTAGATTCTTGTTTGAAACTC** 2100<br>**AAAGAATTCGTAATCATGGTCATAGCTGTTTCCTGTGTGAAATTGTTATCCGCTCACAAT** 2160<br>**TCCACACAACATACGAGCCGGAAGCATAAAGTGTAAAGCCTGGGGTGCCTAATGAGTGAG** 2220<br>**CTAACTCACATTAATTGCGTTGCGCTCACTG** 2251<br>R4 primer
